# Relationship between molecular pathogen detection and clinical disease in febrile children across Europe: a multicentre, prospective observational study

**DOI:** 10.1016/j.lanepe.2023.100682

**Published:** 2023-07-26

**Authors:** Priyen Shah, Marie Voice, Leonides Calvo-Bado, Irene Rivero-Calle, Sophie Morris, Ruud Nijman, Claire Broderick, Tisham De, Irini Eleftheriou, Rachel Galassini, Aakash Khanijau, Laura Kolberg, Mojca Kolnik, Aleksandra Rudzate, Manfred G. Sagmeister, Nina A. Schweintzger, Fatou Secka, Clare Thakker, Fabian van der Velden, Clementien Vermont, Katarina Vincek, Philipp K.A. Agyeman, Aubrey J. Cunnington, Ronald De Groot, Marieke Emonts, Katy Fidler, Taco W. Kuijpers, Marine Mommert-Tripon, Karen Brengel-Pesce, Francois Mallet, Henriette Moll, Stéphane Paulus, Marko Pokorn, Andrew Pollard, Luregn J. Schlapbach, Ching-Fen Shen, Maria Tsolia, Effua Usuf, Michiel van der Flier, Ulrich von Both, Shunmay Yeung, Dace Zavadska, Werner Zenz, Victoria Wright, Enitan D. Carrol, Myrsini Kaforou, Federico Martinon-Torres, Colin Fink, Michael Levin, Jethro Herberg, the PERFORM consortium, Priyen Shah, Marie Voice, Leonides Calvo-Bado, Irene Rivero Calle, Sophie Morris, Ruud Nijman, Claire Broderick, Tisham De, Irini Eleftheriou, Rachel Galassini, Aakash Khanijau, Laura Kolberg, Mojca Kolnik, Aleksandra Rudzate, Manfred Sagmeister, Nina Schweintzger, Fatou Secka, Clare Thakker, Fabian Van der Velden, Clementien Vermont, Katarina Vincek, Philipp K.A. Agyeman, Aubrey J. Cunnington, Ronald De Groot, Marieke Emonts, Katy Fidler, Taco Kuijpers, Marine Mommert-Tripon, Karen Brengel-Pesce, Francois Mallet, Henriette Moll, Stéphane Paulus, Marko Pokorn, Andrew Pollard, Luregn J. Schlapbach, Ching-Fen Shen, Maria Tsolia, Effua Usuf, Michiel Van der Flier, Ulrich Von Both, Shunmay Yeung, Dace Zavadska, Werner Zenz, Victoria Wright, Enitan D. Carrol, Myrsini Kaforou, Federico Martinon-Torres, Colin Fink, Michael Levin, Jethro Herberg, Lucas Baumard, Evangelos Bellos, Lachlan Coin, Giselle D'Souza, Dominic Habgood-Coote, Shea Hamilton, Cllive Hoggart, Sara Hourmat, Heather Jackson, Naomi Lin, Stephanie Menikou, Samuel Nichols, Ivonne Pena Paz, Oliver Powell, Ortensia Vito, Clare Wilson, Amina Abdulla, Ladan Ali, Sarah Darnell, Rikke Jorgensen, Ian Maconochie, Sobia Mustafa, Salina Persand, Ben Walsh, Molly Stevens, Nayoung Kim, Eunjung Kim, Benjamin Pierce, Julia Dudley, Vivien Richmond, Emma Tavliavini, Ching-Chuan Liu, Shih-Min Wang, Fernando Álves González, Cristina Balo Farto, Ruth Barral-Arca, María Barreiro Castro, Xabier Bello, Mirian Ben García, Sandra Carnota, Miriam Cebey-López, María José Curras-Tuala, Carlos Durán Suárez, Luisa García Vicente, Alberto Gómez-Carballa, Jose Gómez Rial, Pilar Leboráns Iglesias, Nazareth Martinón-Torres, José María Martinón Sánchez, Belén Mosquera Pérez, Jacobo Pardo-Seco, Lidia Piñeiro Rodríguez, Sara Pischedda, Sara Ray Vázquez, Carmen Rodríguez-Tenreiro, Lorenzo Redondo-Collazo, Miguel Sadiki Ora, Antonio Sallas, Sonia Serén Fernández, Cristina Serén Trasorras, Marisol Vilas Iglesias, Anda Balode, Arta Bãrdzdina, Dãrta Deksne, Dace Gardovska, Dagne Grãvele, Ilze Grope, Anija Meiere, Ieve Nokalna, Jana Pavãre, Zanda Pučuka, Katrīna Selecka, Dace Svile, Urzula Nora Urbãne, Kalifa Bojang, Syed M.A. Zaman, Suzanne Anderson, Anna Roca, Isatou Sarr, Momodou Saidykhan, Saffiatou Darboe, Samba Ceesay, Umberto D'alessandro, Dorine M. Borensztajn, Nienke N. Hagedoorn, Chantal Tal, Joany Zachariasse, W. Dik, Christoph Aebi, Christoph Berger, Verena Wyss, Mariama Usman, Eric Giannoni, Martin Stocker, Klara M. Posfay-Barbe, Ulrich Heininger, Sara Bernhard-Stirnemann, Anita Niederer-Loher, Christian Kahlert, Giancarlo Natalucci, Christa Relly, Thomas Riedel, Elizabeth Cocklin, Rebecca Jennings, Joanne Johnson, Simon Leigh, Karen Newall, Sam Romaine, Maria Tambouratzi, Antonis Marmarinos, Marietta Xagorari, Kelly Syggelou, Nikos Spyridis, Jennifer Blackmore, Rebekah Harrison, Benno Kohlmaier, Daniela S. Kohlfürst, Christoph Zurl, Alexander Binder, Susanne Hösele, Manuel Leitner, Lena Pölz, Glorija Rajic, Sebastian Bauchinger, Hinrich Baumgart, Martin Benesch, Astrid Ceolotto, Ernst Eber, Siegfried Gallisti, Gunther Gores, Harald Haidl, Almuthe Hauer, Christa Hude, Markus Keldorfer, Larissa Krenn, Heidemarie Pilch, Andreas Pfleger, Klaus Pfurtscheller, Gudrun Nordberg, Tobias Niedrist, Siegfried Rödl, Andrea Skrabl-Baumgartner, Matthias Sperl, Laura Stampfer, Volker Strenger, Holger Till, Andreas Trobisch, Sabine Löffler, Juan Emmanuel Dewez, Martin Hibberd, David Bath, Alec Miners, Elizabeth Fitchett, Catherine Wedderburn, Anne Meierford, Baptiste Leurent, Marien I. De Jonge, Koen van Aerde, Wynand Alkema, Bryan van den Broek, Jolein Gloerich, Alain J. Van Gool, Stefanie Henriet, Martijn Huijnen, Ria Philipsen, Esther Willems, G.P.J.M. Gerrits, M. Van Leur, J. Heidema, L. De Haan, C.J. Miedema, C. Neeleman, C.C. Obihara, G.A. Tramper-Stranders, Rama Kandasamy, Michael J. Carter, Daniel O'Connor, Sagida Bibi, Dominic F. Kelly, Meeru Gurung, Stephen Throson, Imran Ansari, David R. Murdoch, Shrijana Shrestha, Zoe Oliver, Emma Lim, Lucille Valentine, Karen Allen, Kathryn Bell, Adora Chan, Stephen Crulley, Kirsty Devine, Daniel Fabian, Sharon King, Paul McAlinden, Sam McDonald, Anne McDonell, Alisa Pickering, Evelyn Thomson, Amanda Wood, Diane Wallia, Phil Woodsford, Frances Baxter, Ashley Bell, Mathew Rhodes, Rachel Agbeko, Christine Mackerness, Bryan Baas, Lieke Kloosterhuis, Wilma Oosthoek, Tasnim Arif, Joshua Bennet, Kalvin Collings, Ilona Van der Giessen, Alex Martin, Aqeela Rashid, Emily Rowlands, Joshua Soon, Gabriella De Vries, Fabian van der Velden, Mike Martin, Ravi Mistry, Manuela Zwerenz, Judith Buschbeck, Christoph Bidlingmaier, Vera Binder, Katharina Danhauser, Nikolaus Haas, Matthias Griese, Matthias Kappler, Eberhard Lurz, Georg Muench, Karl Reiter, Carola Schoen, Karen Brengel-Pesce, Alexandre Pachot, Marine Mommert, Katarina Vincek, Tina Plankar Srovin, Natalija Bahovec, Petra Prunk, Veronika Osterman, Tanja Avramoska, Ilse Jongerius, J.M. van den Berg, D. Schonenberg, A.M. Barendregt, D. Pajkrt, M. van der Kuip, A.M. van Furth, Evelien Sprenkeler, Judith Zandstra, G. van Mierlo, J. Geissler

**Affiliations:** a Section of Paediatric Infectious Disease, Department of Infectious Diseases, and Centre for Paediatrics and Child Health, Imperial College, London, UK; b Micropathology Ltd, University of Warwick, Coventry, UK; c Translational Pediatrics and Infectious Diseases, Pediatrics Department, Hospital Clínico Universitario de Santiago, Santiago de Compostela, Spain; d GENVIP Research Group, Instituto de Investigación Sanitaria de Santiago, Universidad de Santiago de Compostela, Galicia, Spain; e 2nd Department of Pediatrics, National and Kapodistrian University of Athens, “P. and A. Kyriakou” Children's Hospital, Thivon and Levadias, Goudi, Athens, Greece; f Department of Clinical Infection, Microbiology and Immunology, University of Liverpool Institute of Infection, Veterinary and Ecological Sciences, Liverpool, UK; g Division Paediatric Infectious Diseases, Dr. von Hauner Children's Hospital, University Hospital, LMU Munich, Munich, Germany; h Division of Pediatrics and Department of Infectious Diseases, University Medical Centre Ljubljana, Slovenia; i Children's Clinical University Hospital, Riga, Latvia; j Division of General Pediatrics, Department of Pediatrics and Adolescent Medicine, Medical University of Graz, Graz, Austria; k Medical Research Council Unit The Gambia at LSHTM, Fajara, The Gambia; l Great North Children's Hospital, Paediatric Immunology, Infectious Diseases & Allergy, Newcastle Upon Tyne Hospitals NHS Foundation Trust, UK; m Department of Paediatric Infectious Diseases & Immunology, Erasmus MC-Sophia Children's Hospital, Rotterdam, the Netherlands; n Department of Pediatrics, Inselspital, Bern University Hospital, University of Bern, Switzerland; o Radboud Center for Infectious Diseases, Radboudumc, Nijmegen, the Netherlands and Section Pediatric Infectious Diseases, Laboratory of Medical Immunology, Radboud Institute for Molecular Life Sciences, the Netherlands; p Translational and Clinical Research Institute, Newcastle University, Newcastle upon Tyne, UK; q Royal Alexandra Children's Hospital, Brighton, UK; r Department of Pediatric Immunology, Rheumatology and Infectious Diseases, Amsterdam University Medical Center (AUMC), University of Amsterdam, Amsterdam, the Netherlands; s Sanquin Research Institute, & Landsteiner Laboratory at the AMC, University of Amsterdam, Amsterdam, the Netherlands; t Open Innovation & Partnerships (OIP), bioMérieux S.A., Marcy l'Etoile, France; u Department of General Paediatrics, Erasmus MC-Sophia Children's Hospital, Rotterdam, the Netherlands; v Oxford Vaccine Group, Department of Paediatrics, University of Oxford, Oxford, UK; w Department of Pediatrics, Faculty of Medicine, University of Ljubljana, Slovenia; x Department of Intensive Care and Neonatology, Children's Research Center, University Children's Hospital Zurich, University of Zurich, Zurich, Switzerland; y Department of Paediatrics, National Cheng Kung University Hospital, College of Medicine, National Cheng Kung University, Tainan, Taiwan; z Institute of Clinical Medicine, College of Medicine, National Cheng Kung University, Tainan, Taiwan; aa Pediatric Infectious Diseases and Immunology, Wilhelmina Children's Hospital, University Medical Center Utrecht, Utrecht, the Netherlands; ab German Center for Infection Research (DZIF), Partner Site Munich, Munich, Germany; ac Faculty of Infectious and Tropical Disease, London School of Hygiene and Tropical Medicine, London, UK; ad Riga Stradins University, Riga, Latvia; ae Department of Infectious Diseases, Alder Hey Children's Hospital, Eaton Road, Liverpool, UK; af Centro de Investigación Biomédica en Red de Enfermedades Respiratorias (CIBERES), Instituto de Salud Carlos III, Madrid, Spain

**Keywords:** Molecular diagnostics, Diagnostic, Febrile illness, Infectious disease, Bacterial, Viral, Respiratory infection

## Abstract

**Background:**

The PERFORM study aimed to understand causes of febrile childhood illness by comparing molecular pathogen detection with current clinical practice.

**Methods:**

Febrile children and controls were recruited on presentation to hospital in 9 European countries 2016–2020. Each child was assigned a standardized diagnostic category based on retrospective review of local clinical and microbiological data. Subsequently, centralised molecular tests (CMTs) for 19 respiratory and 27 blood pathogens were performed.

**Findings:**

Of 4611 febrile children, 643 (14%) were classified as definite bacterial infection (DB), 491 (11%) as definite viral infection (DV), and 3477 (75%) had uncertain aetiology. 1061 controls without infection were recruited. CMTs detected blood bacteria more frequently in DB than DV cases for *N. meningitidis* (OR: 3.37, 95% CI: 1.92–5.99), *S. pneumoniae* (OR: 3.89, 95% CI: 2.07–7.59), Group A streptococcus (OR 2.73, 95% CI 1.13–6.09) and *E. coli* (OR 2.7, 95% CI 1.02–6.71). Respiratory viruses were more common in febrile children than controls, but only influenza A (OR 0.24, 95% CI 0.11–0.46), influenza B (OR 0.12, 95% CI 0.02–0.37) and RSV (OR 0.16, 95% CI: 0.06–0.36) were less common in DB than DV cases. Of 16 blood viruses, enterovirus (OR 0.43, 95% CI 0.23–0.72) and EBV (OR 0.71, 95% CI 0.56–0.90) were detected less often in DB than DV cases. Combined local diagnostics and CMTs respectively detected blood viruses and respiratory viruses in 360 (56%) and 161 (25%) of DB cases, and virus detection ruled-out bacterial infection poorly, with predictive values of 0.64 and 0.68 respectively.

**Interpretation:**

Most febrile children cannot be conclusively defined as having bacterial or viral infection when molecular tests supplement conventional approaches. Viruses are detected in most patients with bacterial infections, and the clinical value of individual pathogen detection in determining treatment is low. New approaches are needed to help determine which febrile children require antibiotics.

**Funding:**

EU Horizon 2020 grant 668303.

**Research in context**

**Evidence before this study**

We searched PubMed and MedLine databases on July 30, 2021 using the search terms (“molecular” OR “pcr”) AND (“infection” OR “sepsis” OR “bacterial” OR “viral”) AND “diagnosis”. We limited our search to studies published after July 30, 2011 with patients under 18 years where pathogen detection was used to ascertain the cause of infection or the need for treatment. All studies identified were either limited to a specific site of infection (pneumonia, meningitis, diarrhoeal illness etc) and/or focused on a specific class of pathogen (bacteria, viruses, respiratory pathogens, gut pathogens etc). We found no studies that applied both bacterial and viral molecular diagnostics to patients with a variety of infectious illnesses, including patients where the source or cause of infection was not clear. The current literature presents conflicting reports on the utility of molecular diagnostics to identify the etiology of a febrile illness and identify patients who require antibiotic treatment.

**Added value of this study**

The PERFORM study prospectively recruited febrile children (FC) from emergency departments, inpatient wards and intensive care units at 17 hospitals in 9 European countries. In addition to detailed clinical, laboratory and imaging data, centralized molecular tests (CMT) were performed on blood and/or throat swabs from 4611 patients with confirmed bacterial or viral infection, or an unclear cause of infection and 1061 controls.

By performing CMTs for all 25 viral, 27 bacterial and 6 fungal targets on all patients with available samples, regardless of clinical assignment and whether the cause of infection was known, we are able to present an in-depth overview of the pathogens detected in FC across Europe at the point of presentation to healthcare services.

The results challenge the appropriateness of defining febrile illness into that caused by bacteria and that caused by viruses. Despite best practice investigation at each participating hospital, only 14% of FC with suspected infection were assigned as having definite bacterial infection, 11% as definite viral infection, and the remaining 75% had an uncertain diagnosis. The addition of CMTs increased the numbers of pathogens identified, but did not significantly improve diagnostic assignment, as viruses were identified in blood or throat swabs of a high proportion of FC with confirmed bacterial infections.

**Implications of all the available evidence**

Our study suggests that a reliance on pathogen detection to diagnose and manage febrile children is largely unsuccessful, and has limited value for clinical decision-making as to which patients are likely to have severe bacterial infections and thus which should receive antibiotics.

Identification of a virus in samples from a febrile patient offers little reassurance that they do not have a bacterial infection, and our data support the observation that viral infections frequently precede or contribute to bacterial infections. Our study suggests that new approaches other than pathogen detection will be needed to address the question of which children require antibiotics.

## Introduction

Fever is among the commonest cause of patients seeking medical care in both hospital and community settings.^1^^,^^2^ Most patients with fever, who seek medical care, suffer from self-resolving infections.^3^, ^4^, ^5^, ^6^ However, amongst them are a smaller group with bacterial infection, including severe infections such as pneumonia, sepsis and meningitis, who require antibiotic treatment.^3^^,^^7^

Clinical features do not reliably distinguish bacterial infection from common self-limiting viral infections.^8^^,^^9^ Delays in diagnosis of serious bacterial infections such as sepsis, meningitis and pneumonia can lead to death or disability.^10^^,^^11^ As infection remains the leading cause of death in young children,^12^^,^^13^ the usual approach to management has focused on “ruling out sepsis”. This often involves the child undergoing blood and urine cultures, lumbar puncture, and imaging. As results of microbiological cultures are not available for 24–48 hours, patients who appear ill are commenced on antibiotics while awaiting culture results.^14^ This approach results in investigation, admission, and treatment with antibiotics of innumerable children worldwide, only a small proportion of whom have or will develop invasive bacterial infections. It is associated with considerable resource use, and cost to families and health services, and potentially contributes to development of antimicrobial resistance.^15^^,^^16^

Molecular methods can detect the DNA or RNA of bacterial, viral, fungal, and parasitic pathogens rapidly,^17^, ^18^, ^19^, ^20^, ^21^, ^22^ and the medical and biotechnology community has invested considerable effort and resources in the hope that rapid pathogen detection by molecular methods would transform diagnosis and management of patients with suspected infection.^23^, ^24^, ^25^, ^26^, ^27^, ^28^, ^29^ The PERFORM study evaluated diagnosis of febrile children attending hospital using current best local practice hospital diagnostics supplemented by centralized research molecular pathogen detection tests (CMT). This study on the relationship between molecular pathogen detection and clinical disease had 3 key objectives:

1. Describe the pathogens detected by CMT in blood and respiratory samples of children presenting with a febrile illness or suspected infection across Europe.
2. Establish whether pathogen detection, when incorporating highly specific CMT results, can be used to determine etiology or inform management of a febrile illnesses in children.
3. Explore whether certain pathogens are more likely to be detected in patients with bacterial or viral disease.

## Methods

### Study design

PERFORM (https://www.perform2020.org) is an EU-funded multi-country study that prospectively recruited febrile children at 17 hospitals in 9 European countries between April 2016 and December 2020. Study sites and countries involved are shown in Table S1. Children aged under 18 years of age presenting with fever, history of fever during their presenting illness, or suspected infection who were considered ill enough to warrant blood tests, were recruited from emergency departments, pediatric inpatient wards, and intensive care units. Non-febrile children attending hospital with non-infectious conditions, who were undergoing blood sampling for other reasons were opportunistically recruited as controls if they had no febrile symptoms or vaccinations within the previous 3 weeks.

Recruited febrile children (FC) underwent clinical assessment and investigation as part of their clinical care, according to routine practice at each institution, including blood and urine cultures, microscopy, and cultures of cerebrospinal fluid (where clinically indicated), and detection of bacteria and viruses in throat swabs or nasopharyngeal aspirates. Serological studies, routine molecular tests (where available and indicated), and chest radiographs and other imaging were undertaken at the discretion of treating clinicians. Serial clinical findings and results from all investigations were recorded on an electronic standardized case report form and stored on a centralized database. The study was conducted following the defined study protocol (Supplementary Methods).

### Clinical definitions and assignment

**Fig. 1:**
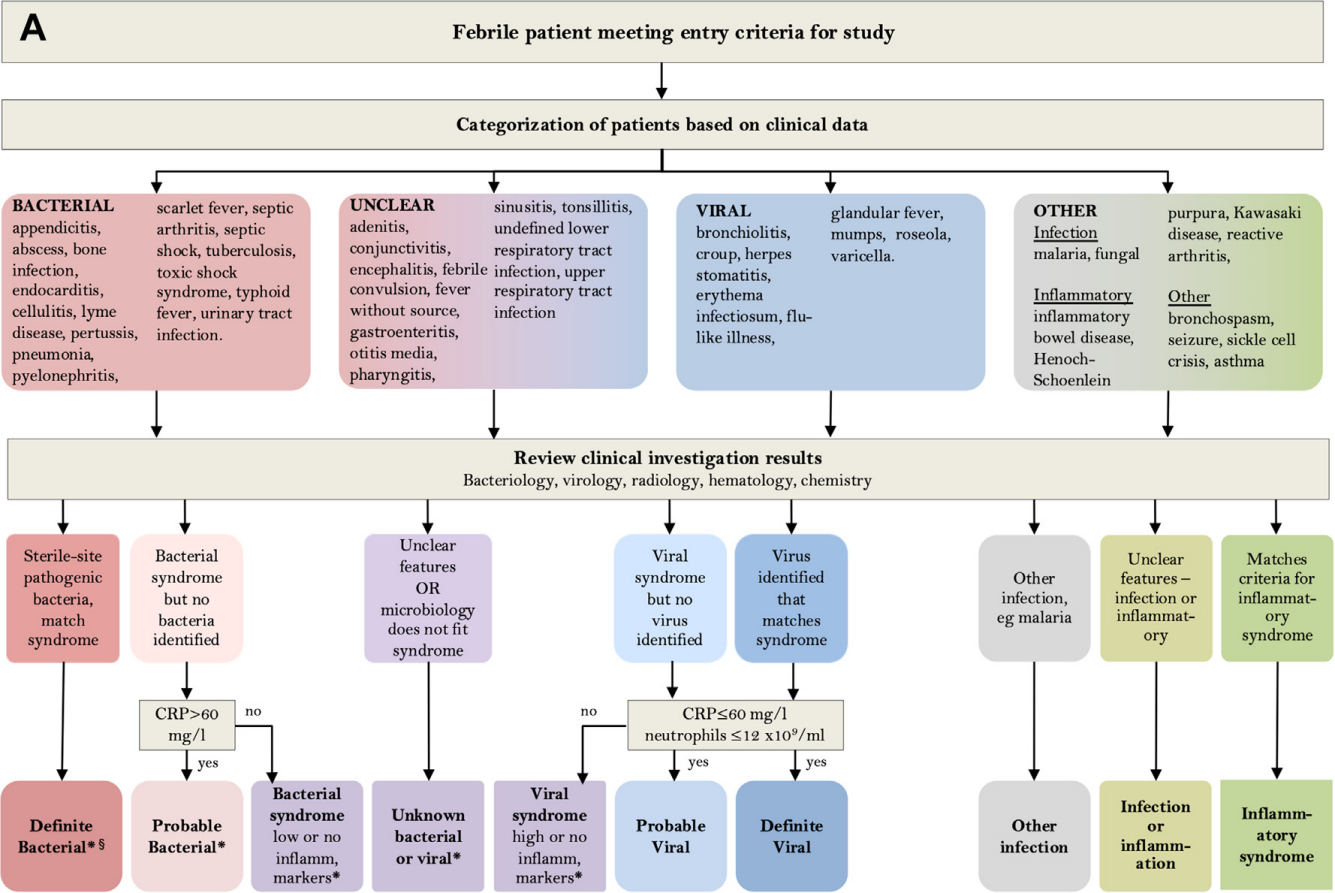

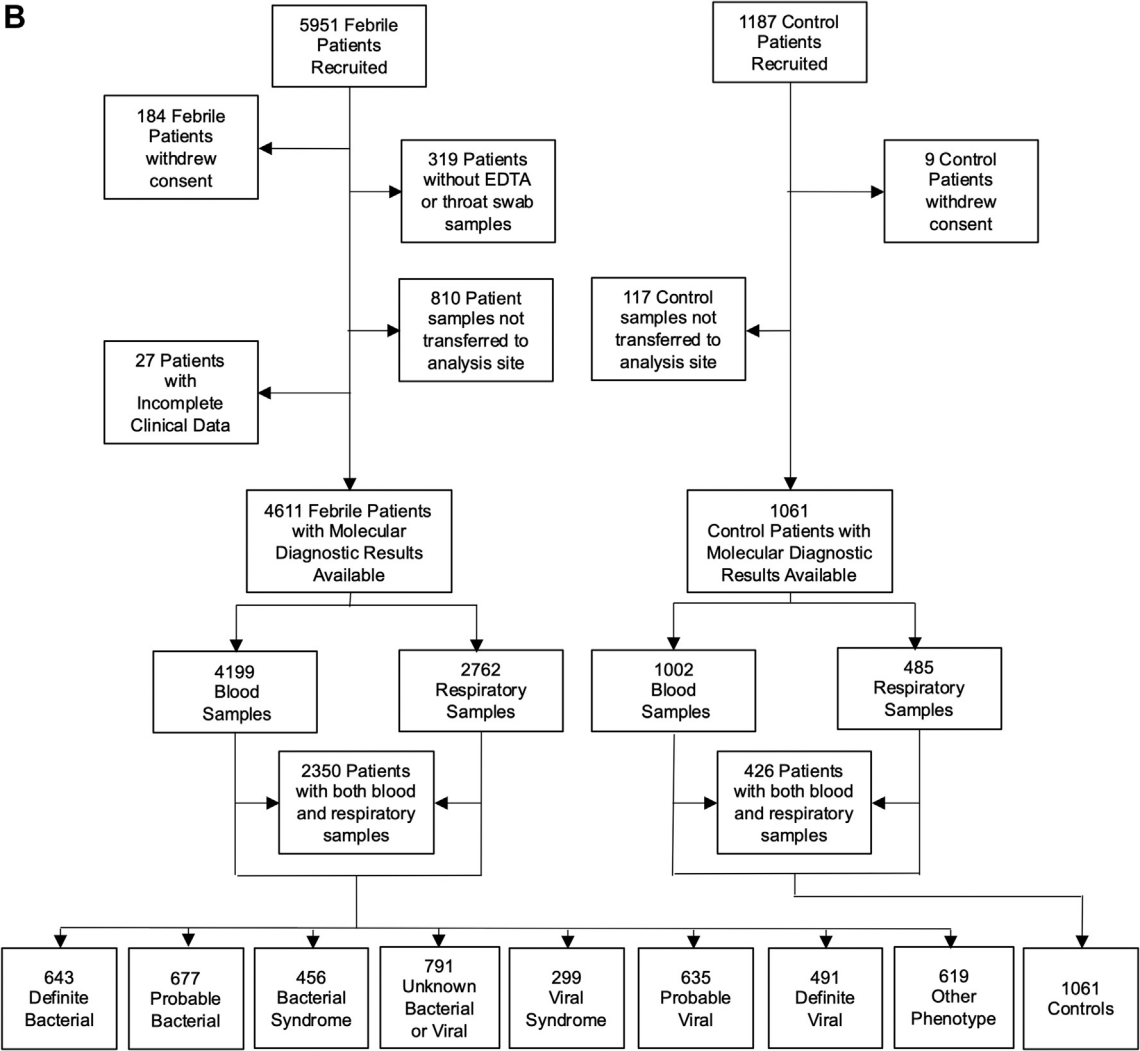

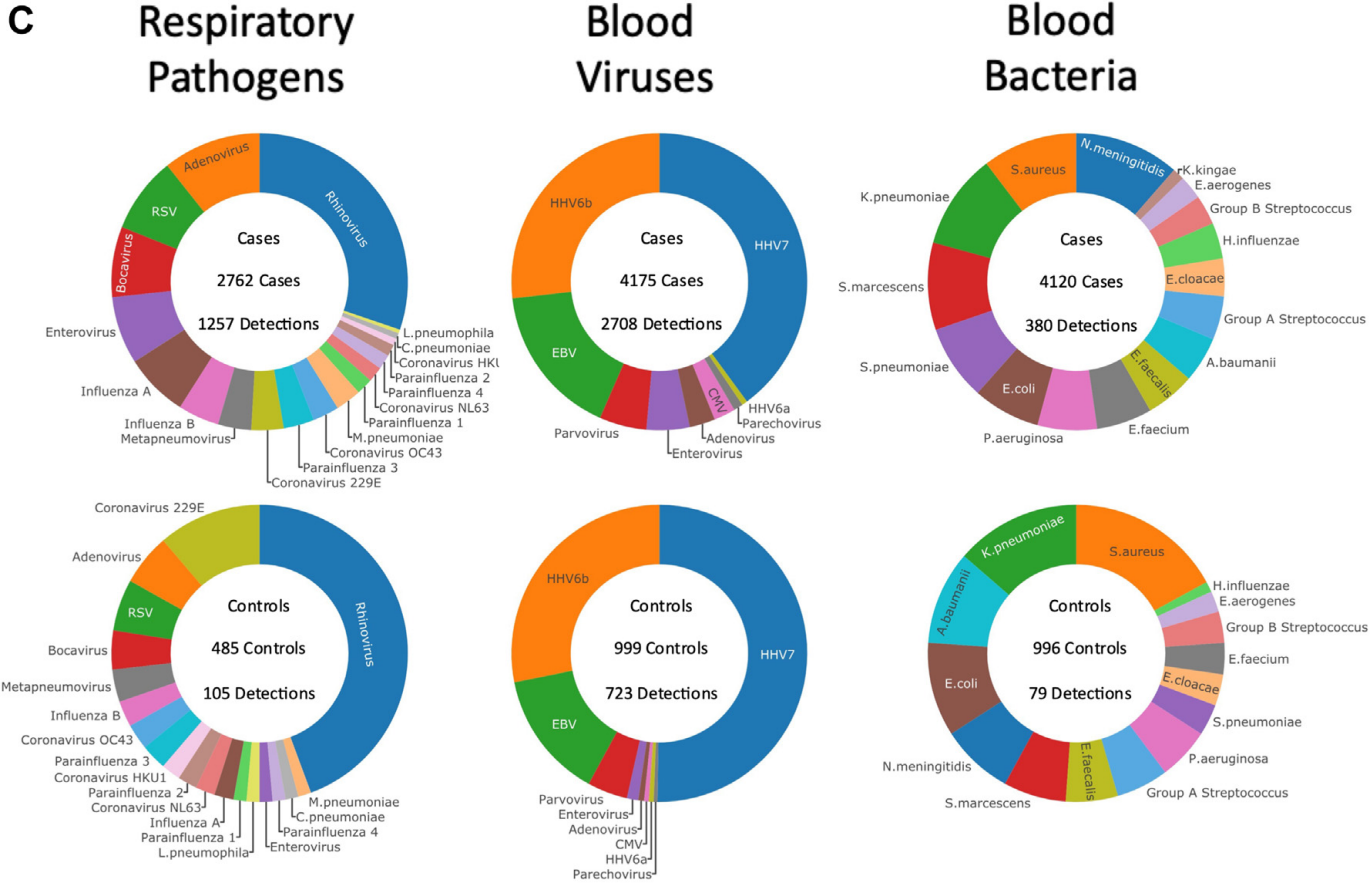
(A) Clinical investigative process to assign patients to individual diagnostic categories. ∗Denotes diagnostic categories in which viral co-infection may or may not be present. ^§^Pathogens normally only detected on mucosal surfaces or diagnosed serologically (*M. tuberculosis*, *B. pertussis*, *M. pneumoniae*, *Borrelia* species, *Campylobacter* and *Salmonella)* were included in the Definite Bacterial category if the clinical presentation was consistent with the pathogen detected, but were analyzed separately for some analyses. (B) Patient flow and numbers from recruitment to final diagnostic categories. Children with fever, history of recent fever or suspicion of infection of sufficient severity to warrant blood tests were assigned to a presumptive diagnostic group based on clinical findings on presentation. After results of all microbiological investigations, biochemical and hematological investigations and imaging were available, patients were assigned to final diagnostic categories using the definitions in Table 1. (C) Frequency of detection of pathogen DNA or RNA by research molecular tests in cases and controls. Donut plots show the breakdown of the total number of patients with one or more positive molecular pathogen detection from the research study in cases and controls for respiratory pathogens found in throat swabs, viruses detected in blood, and bacteria detected in blood.

A final diagnostic category was assigned for each patient, after review by at least two experienced pediatricians of all clinical, laboratory and imaging data at each hospital, based on the diagnostic approach in Fig. 1A and definitions in Table 1. Centralized molecular pathogen Tests (CMT) were undertaken retrospectively on all patient samples, in order to generate a comprehensive view of the pathogens present, irrespective of the clinical syndrome described for each patient. Results were not used for clinical management or for local assignment by the research team into diagnostic categories.

**Table 1:** Definitions used to assign patients to diagnostic categories.

Diagnostic Categories	Definition
Definite Bacterial infection (DB)	Patients in whom an appropriate bacterial pathogen is isolated from a normally sterile site, and clinical presentation is consistent with the pathogen isolated. Detection of virus does not influence assignment. Pathogens normally only detected on mucosal surfaces or diagnosed serologically (*M. tuberculosis*, *B. pertussis*, *M. pneumoniae*, *Borrelia* species, *Campylobacter* and *Salmonella)* can be included if clinical presentation consistent with pathogen detected, but are analyzed separately for some analyses.
Probable Bacterial infection (PB)	Clinical features consistent with bacterial infection (e.g. lobar pneumonia, empyema, septic arthritis, osteomyelitis, cellulitis, meningitis) with elevated inflammatory markers (maximum CRP > 60 mg/L and white cell count >12 × 10^−9^/L). No bacterial pathogen is identified at a sterile site. Bacteria may or may not be identified at non-sterile sites (mucosal surfaces, stool). Detection of a virus does not alter assignment.
Bacterial syndrome	Clinical features consistent with a bacterial infection, with no sterile site bacterial pathogen identified. Inflammatory markers not elevated (maximum CRP ≤ 60 mg/L) or not measured. Bacteria may or may not be identified at non-sterile sites. Virus detection does not alter assignment.
Unknown Bacterial or Viral Infection	Patients with a presumed infection whose clinical features do not clearly indicate a bacterial or a viral cause, or in whom the available microbiological results are inconsistent with the clinical syndrome.
Viral Syndrome	Clinical features consistent with a viral infection (such as bronchiolitis, varicella, or measles), in whom a virus may or may not be identified, and with inflammatory markers that are high (maximum CRP > 60 mg/L or neutrophil count >12 × 10^9^ cells/ml) or were not measured. No sterile-site bacteria identified.
Probable Viral infection (PV)	Clinical features consistent with a viral infection, with no viral pathogen identified that is consistent with all features of the illness. Maximum CRP ≤ 60 mg/L and neutrophil count ≤ 12 × 10^9^ cells/ml. No sterile-site bacteria are identified.
Definite Viral infection (DV)	Patients with a viral pathogen isolated that is likely to account for all features of the illness. Maximum CRP ≤ 60 mg/L and neutrophil count ≤12 × 10^9^ cells/ml. No sterile-site bacteria identified.
Other infection	Patients with non-bacterial, non-viral pathogens such as malaria, and fungi.
Other phenotype	Patients with a non-infectious disease including patients with inflammatory diseases such as Kawasaki disease and other illnesses such as seizures or non-infectious exacerbations of asthma

### Centralized molecular pathogen tests (CMT)

Research blood and respiratory samples were collected as early as possible in the patients’ hospital admission, where possible at the same time as the first clinically indicated blood tests. A dry flocked throat swab stored in eNAT® and blood collected in EDTA were stored at −80 °C and transported to Micropathology Ltd, University of Warwick, Coventry, U.K.

Total nucleic acid was extracted from the throat swab and blood samples. Throat swabs were analyzed by the NxTAG™ Respiratory Pathogen Panel (RPP) assay (Luminex® Corporation), enabling the simultaneous detection of 16 viral and 3 bacterial species (Table S2). For blood samples, a panel of in-house molecular diagnostic tests was developed and validated (Supplementary Methods) at Micropathology Ltd for the PERFORM study which included the common or clinically significant childhood pathogens. This comprised a panel of 14 multiplex probe-based qPCR assays, comprising 9 viral targets (Table S3), 24 bacterial targets and 6 fungal targets (Table S4), enabling the detection of 9 viral, 16 bacterial and 2 fungal species from whole blood extracts (Supplementary Methods). Results of these CMTs are reported separately from local investigations which may also include molecular testing for common pathogens.

### Analysis and statistics

Clinical and laboratory data collected by local investigators, and the locally assigned diagnostic categories were entered on a secure, online custom-built database, with personal identifiers removed. All patients were assigned to definite viral infection (DV), definite bacterial infection (DB) or unknown bacterial or viral diagnostic categories, and 10% of all other assignments were cross-checked for inconsistencies by external monitors from a different site or by the central study team.

The results from CMTs were assessed in relation to the locally assigned diagnostic categories, by calculating odds ratios (ORs) with 95% confidence intervals (CIs) for the detection of each organism in both bacterial vs viral diagnostic categories, and in cases vs controls. We used a multiple logistic regression model with iteratively re-weighted least squares using the R package glmnet.^30^ Age, gender, ethnicity, country of recruitment, immunodeficiency status and clinical syndrome were investigated as potential confounding factors (Table S5). Age was identified as the only significant factor and the logistic regression model was therefore designed with age as a covariate. In addition, to reflect the differences in epidemiology and investigation of febrile illnesses in infants compared to older children, ORs with 95% CIs were calculated for the detection of each organism in cases vs controls and bacterial vs viral diagnostic categories in children under 1 and children over 1 separately. For blood viral assays, where pathogen load counts were available, ORs with 95% CIs were calculated at increasing quartiles of viral load for the detection of each virus in cases vs controls, and in definite bacterial vs viral diagnostic categories. The ability of a positive virus test to exclude a bacterial infection was calculated for each individual virus, and for the aggregated detection of any virus in blood or respiratory samples. All analyses were conducted in R Programming version 4.0.2.^31^

### Ethics and study governance

Ethical approval was obtained at the coordinating site (Imperial College London, 16/LO/1684) and separately at each participating center (see supplement), using a consortium-wide clinical study protocol (Supplementary Methods). All patients were recruited with informed parental consent, and assent from older children. PERFORM membership and roles in study design, data collection and analysis are described in the Supplementary Appendix. The initial manuscript was drafted by the corresponding author and developed by all members of the writing group. The corresponding author and analysis group had access to all data, vouching for the completeness and accuracy of data.

### Role of the funding source

PERFORM was funded by the European Union's Horizon 2020 program under GA No 668303. Enrolment of patients in the UK was supported by NIHR Biomedical Research Centres at Imperial College London, and Newcastle (see Supplementary). The funding source had no role in study design; in the collection, analysis, and interpretation of data; in the writing of the manuscript; or in the decision to submit the paper for publication.

## Results

From April 2016 to December 2019, 5951 patients and 1187 controls were enrolled. After removal of patients with no available research samples or incomplete data, 4611 patients were included in the analysis. 4199 had molecular pathogen detection on blood, 2762 on throat swabs, and 2350 both blood and throat swabs available (Fig. 1B). The diagnostic categories assigned to the 4611 patients by the local recruiting team, using all clinical and local investigation data, comprised 643 patients with DB phenotype (14%), and 491 with DV phenotype (11%), with the remaining 3477 (75%) assigned to probable bacterial or viral (PB/PV), or to other less certain infection or inflammation categories (Fig. 1B). 1061 controls were included of which 1002 had molecular pathogen detection on blood, 485 on throat swabs and 426 on both. The clinical details of the patients assigned to each diagnostic group are shown in Table 2. A high proportion of children received antibiotics, including those whose final diagnosis was DV or PV infection (95% (613/643) for DB, 99% (671/677) for PB, 71% (348/491) for DV and 48% (306/635) for PV).

**Table 2:** Clinical characteristics of patients.

	Definite bacterial (n = 643)	Probable bacterial (n = 677)	Bacterial syndrome (n = 456)	Unknown bacterial or viral (n = 791)	Viral syndrome (n = 299)	Probable viral (n = 635)	Definite viral (n = 491)	Other phenotype (n = 619)	Control (n = 1061)	Overall (n = 5672)
**Gender M (%)**	348 (54.1%)	375 (55.4%)	253 (55.5%)	453 (57.3%)	175 (58.5%)	352 (55.4%)	262 (53.4%)	340 (54.9%)	601 (56.6%)	3159 (55.7%)
**Age (months)**										
Median [IQR]	58.9 [15.0–131]	59.9 [26.5–128]	63.3 [26.2–128]	55.2 [19.8–111]	38.3 [16.1–86.5]	43.3 [14.8–96.3]	39.7 [9.32–86.4]	104 [38.9–161]	122 [63.2–167]	64.5 [22.7–134]
**Ethnicity**										
Afro Caribbean	1 (0.2%)	1 (0.1%)	3 (0.7%)	6 (0.8%)	1 (0.3%)	4 (0.6%)	2 (0.4%)	8 (1.3%)	3 (0.3%)	29 (0.5%)
Asian	38 (5.9%)	31 (4.6%)	16 (3.5%)	65 (8.2%)	22 (7.4%)	33 (5.2%)	46 (9.4%)	37 (6.0%)	34 (3.2%)	322 (5.7%)
Black African	13 (2.0%)	17 (2.5%)	5 (1.1%)	38 (4.8%)	11 (3.7%)	12 (1.9%)	17 (3.5%)	30 (4.8%)	15 (1.4%)	158 (2.8%)
Middle-Eastern	15 (2.3%)	11 (1.6%)	14 (3.1%)	21 (2.7%)	9 (3.0%)	12 (1.9%)	20 (4.1%)	16 (2.6%)	16 (1.5%)	134 (2.4%)
White European	536 (83.4%)	559 (82.6%)	393 (88.2%)	598 (75.6%)	230 (76.9%)	527 (83.0%)	366 (74.5%)	467 (75.4%)	911 (85.9%)	4587 (80.9%)
Mixed	10 (1.6%)	14 (2.1%)	7 (1.5%)	19 (2.4%)	10 (3.3%)	13 (2.0%)	16 (3.3%)	9 (1.5%)	17 (1.6%)	115 (2.0%)
Other	30 (4.7%)	44 (6.5%)	18 (3.9%)	44 (5.6%)	16 (5.4%)	34 (5.4%)	24 (4.9%)	52 (8.4%)	65 (6.1%)	327 (5.8%)
**Country**										
Austria	44 (6.8%)	77 (11.4%)	33 (7.2%)	64 (8.1%)	14 (4.7%)	19 (3.0%)	42 (8.6%)	34 (5.5%)	165 (15.6%)	492 (8.7%)
Germany	7 (1.1%)	21 (3.1%)	4 (0.9%)	17 (2.1%)	11 (3.7%)	15 (2.4%)	5 (1.0%)	21 (3.4%)	13 (1.2%)	114 (2.0%)
Greece	72 (11.2%)	37 (5.5%)	9 (2.0%)	88 (11.1%)	23 (7.7%)	130 (20.5%)	59 (12.0%)	47 (7.6%)	35 (3.3%)	500 (8.8%)
Latvia	71 (11.0%)	106 (15.7%)	52 (11.4%)	37 (4.7%)	13 (4.3%)	100 (15.7%)	43 (8.8%)	31 (5.0%)	131 (12.3%)	584 (10.3%)
Netherlands	96 (14.9%)	78 (11.5%)	57 (12.5%)	54 (6.8%)	49 (16.4%)	116 (18.3%)	50 (10.2%)	102 (16.5%)	114 (10.7%)	716 (12.6%)
Slovenia	52 (8.1%)	62 (9.2%)	27 (5.9%)	16 (2.0%)	25 (8.4%)	36 (5.7%)	17 (3.5%)	19 (3.1%)	23 (2.2%)	277 (4.9%)
Spain	79 (12.3%)	67 (9.9%)	57 (12.5%)	121 (15.3%)	30 (10.0%)	54 (8.5%)	68 (13.8%)	47 (7.6%)	218 (20.5%)	741 (13.1%)
Switzerland	16 (2.5%)	13 (1.9%)	18 (3.9%)	16 (2.0%)	12 (4.0%)	19 (3.0%)	2 (0.4%)	15 (2.4%)	10 (0.9%)	121 (2.1%)
Taiwan	20 (3.1%)	0 (0%)	0 (0%)	7 (0.9%)	1 (0.3%)	2 (0.3%)	3 (0.6%)	3 (0.5%)	0 (0%)	36 (0.6%)
U.K.	186 (28.9%)	216 (31.9%)	199 (43.6%)	371 (46.9%)	121 (40.5%)	144 (22.7%)	202 (41.1%)	300 (48.5%)	352 (33.2%)	2091 (36.9%)
**Max CRP (mg/L)**										
Median [IQR]	102 [36.4–202]	146 [100–220]	20.5 [7.00–37.0]	35.9 [13.1–79.4]	67.0 [23.3–101]	10.0 [4.00–24.0]	10.3 [4.00–26.0]	19.5 [4.00–78.0]	NA	35.0 [9.00–102]
**Max neutrophils (10ˆ9/L)**										
Median [IQR]	10.2 [5.87–15.4]	12.8 [8.55–18.9]	7.09 [4.52–11.4]	6.68 [3.50–11.3]	12.5 [7.50–15.3]	4.92 [2.89–7.80]	4.46 [2.53–7.20]	6.61 [3.81–11.2]	NA	7.55 [4.10–12.4]
**Required admission**	514 (79.9%)	558 (82.4%)	351 (77.0%)	534 (67.5%)	214 (71.6%)	266 (41.9%)	350 (71.3%)	427 (69.0%)	232 (21.9%)	3446 (60.8%)
**PICU admission**	129 (20.1%)	118 (17.4%)	25 (5.5%)	116 (14.7%)	58 (19.4%)	24 (3.8%)	47 (9.6%)	109 (17.6%)	35 (3.3%)	661 (11.7%)
**Ventilated**	62 (9.6%)	78 (11.5%)	12 (2.6%)	93 (11.8%)	47 (15.7%)	15 (2.4%)	39 (7.9%)	64 (10.3%)	0 (0%)	410 (7.2%)
**Primary or secondary immunodeficiency**	69 (10.7%)	62 (9.2%)	27 (5.9%)	160 (20.2%)	20 (6.7%)	40 (6.3%)	51 (10.4%)	93 (15.0%)	57 (5.4%)	579 (10.2%)
**Upper respiratory tract infection**	46 (7.2%)	169 (25.0%)	83 (18.2%)	213 (26.9%)	88 (29.4%)	289 (45.5%)	105 (21.4%)	39 (6.3%)	0 (0%)	1032 (18.2%)
**Lower respiratory tract infection**	97 (15.1%)	242 (35.7%)	83 (18.2%)	170 (21.5%)	93 (31.1%)	88 (13.9%)	103 (21.0%)	27 (4.4%)	0 (0%)	903 (15.9%)
**Meningitis or encephalitis**	42 (6.5%)	5 (0.7%)	3 (0.7%)	21 (2.7%)	17 (5.7%)	15 (2.4%)	59 (12.0%)	10 (1.6%)	0 (0%)	172 (3.0%)
**Sepsis**	157 (24.4%)	17 (2.5%)	3 (0.7%)	9 (1.1%)	2 (0.7%)	0 (0%)	0 (0%)	1 (0.2%)	0 (0%)	189 (3.3%)
**Musculoskeletal infection**	33 (5.1%)	11 (1.6%)	21 (4.6%)	7 (0.9%)	0 (0%)	11 (1.7%)	13 (2.6%)	29 (4.7%)	0 (0%)	125 (2.2%)
**Soft tissue infection**	55 (8.6%)	92 (13.6%)	174 (38.2%)	27 (3.4%)	3 (1.0%)	13 (2.0%)	7 (1.4%)	12 (1.9%)	0 (0%)	383 (6.8%)
**Gastrointestinal infection**	84 (13.1%)	62 (9.2%)	47 (10.3%)	62 (7.8%)	28 (9.4%)	69 (10.9%)	44 (9.0%)	29 (4.7%)	0 (0%)	425 (7.5%)
**Urinary tract infection**	204 (31.7%)	55 (8.1%)	12 (2.6%)	11 (1.4%)	2 (0.7%)	0 (0%)	0 (0%)	3 (0.5%)	0 (0%)	287 (5.1%)
**Other diagnosis**	81 (12.6%)	34 (5.0%)	39 (8.6%)	40 (5.1%)	50 (16.7%)	82 (12.9%)	160 (32.6%)	72 (11.6%)	0 (0%)	558 (9.8%)
**Received antibiotics**	613 (95.3%)	671 (99.1%)	438 (96.1%)	631 (79.8%)	196 (65.6%)	306 (48.2%)	348 (70.9%)	357 (57.7%)	0 (0%)	3560 (62.8%)

Ethnicity recorded was the self-reported ethnicity. Maximum CRP and neutrophil count refer to the highest value for each patient during that illness episode. The control group included hospitalized patients with no fever and with conditions considered to be not due to infection or inflammatory disorders. Controls included 35 patients with critical illness and 57 patients with primary or secondary immunodeficiency.

PICU: pediatric intensive care unit; numbers show the median and interquartile range (IQR); CRP: C-reactive protein.

### Pathogens detected by local investigations and by centralized molecular pathogen tests

The spectrum of pathogens detected by local investigations and CMT in all diagnostic groups are shown in Tables S6–S7 (local), and Tables S8–S10 (for CMT). The most frequent CMT identifications for FC were, in decreasing order, rhinovirus, adenovirus and RSV (respiratory pathogens); HHV7, HHV6b and EBV (blood viruses); and *N. meningitidis*, *S. aureus* and *K. pneumoniae* (blood bacteria) (Fig. 1C).

### CMT in febrile cases vs controls

#### Respiratory viruses

**Fig. 2:**
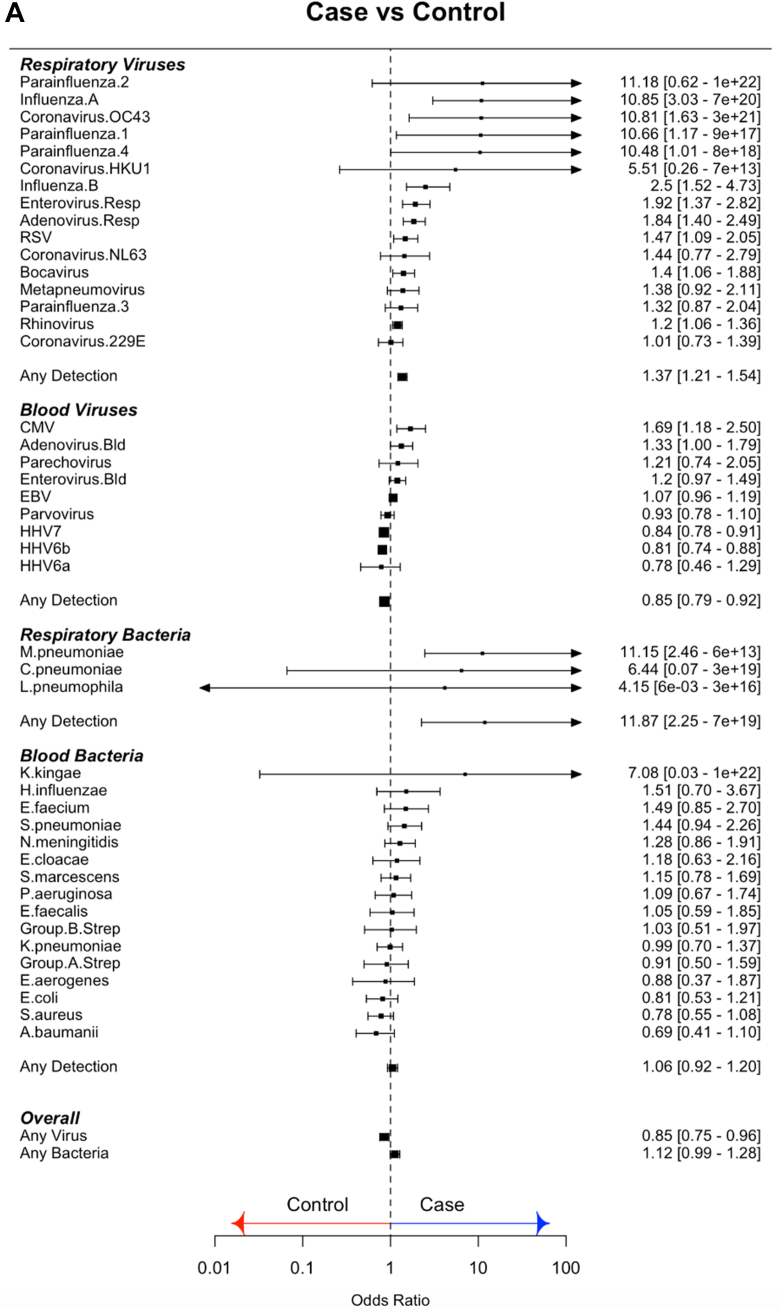

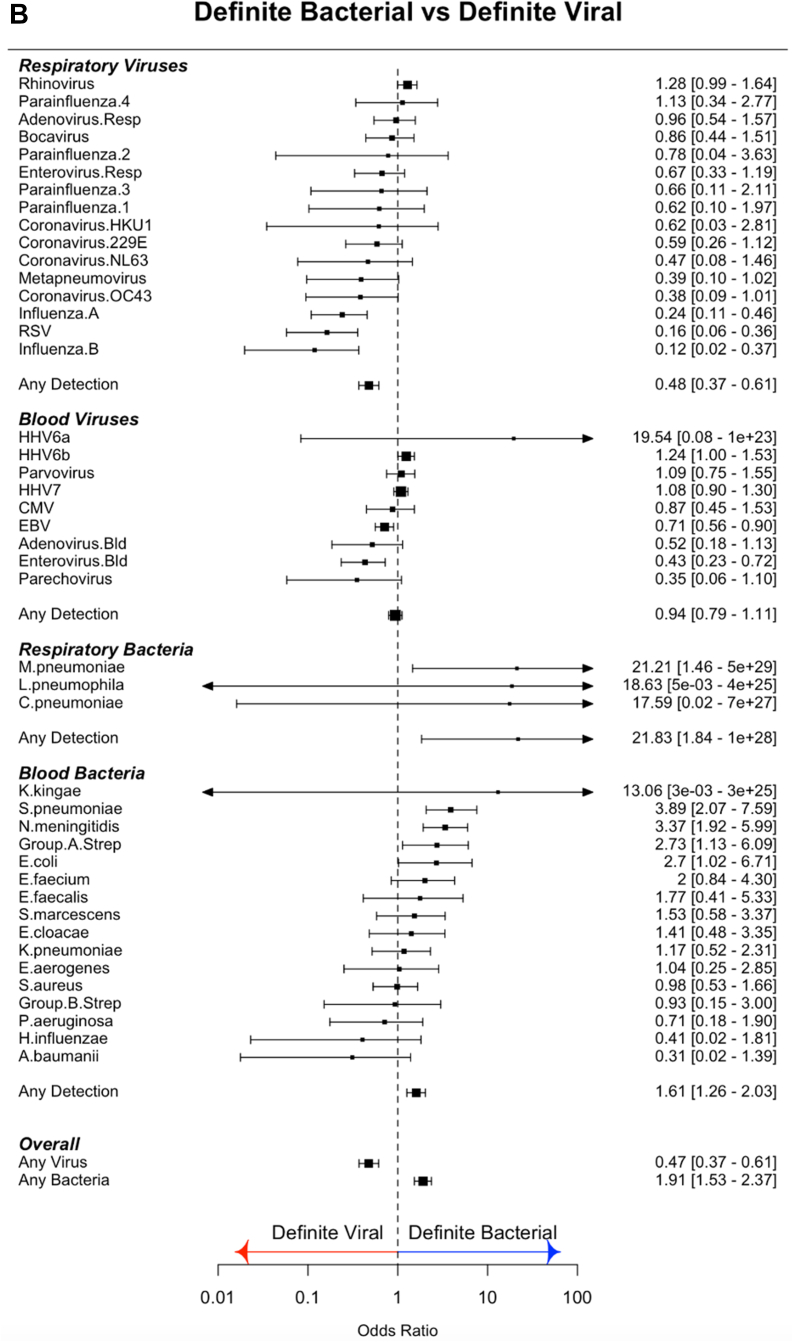
(A) Odds ratios for molecular pathogen identifications comparing febrile children and non-febrile controls. Square symbols show the mean and bars and whiskers the 95% confidence intervals. Odds Ratio's above 1 (to right of the dotted line; blue arrow) indicate increased detection of pathogen in cases. OR less than 1 (left of dotted line; red arrow) indicate increased pathogen detection in controls. (B) Odds ratios for molecular pathogen identifications comparing definite bacterial and definite viral groups. Square symbols show the mean and bars and whiskers the 95% confidence intervals. Odds Ratio's above 1 (to right of the dotted line; blue arrow) indicate increased detection of pathogen in DB; OR less than 1 (left of dotted line; red arrow) indicate increased pathogen detection in the viral group.

1257 of 2762 cases (46%) and 105 of 485 (22%) controls had at least one virus detected in throat swabs (Fig. 1C). Viruses were more commonly detected in FC than controls (OR 1.37 95% CI: 1.21–1.54). Compared to controls, FC had increased frequency of influenza viruses A and B, parainfluenza 1 and 4, bocavirus, rhinovirus, coronavirus OC43, RSV, enterovirus, and adenovirus (Fig. 2A; Table S8).

#### Blood viruses

Viruses were detected in blood less commonly in cases than in controls (cases: 2708/4175 (65%); controls: 723/999 (72%); OR: 0.85, 95% CI: 0.79–0.92). Only CMV and adenovirus were more commonly detected in cases than controls, while HHV7 and HHV6b were more common in controls (Fig. 2A; Table S9).

#### Blood bacteria

Bacterial DNA was detected in blood at low frequency in both cases and controls (cases: 380/4120 (9%); controls: 79/996 (8%); OR 1.06, 95% CI 0.92–1.20). Odds ratios were overlapping for detecting all bacterial targets in cases and controls (Fig. 2A; Table S10).

### CMT in definite bacterial vs definite viral groups

We compared pathogen detection by CMT in the patients assigned a DB or a DV phenotype, to investigate which molecular identifications were reliable predictors of a bacterial or a viral illness.

#### Respiratory pathogens

Influenza A and B, and RSV were more commonly detected in DV than DB, whilst *Mycoplasma pneumoniae* was more common in DB (Fig. 2B). When the Probable Bacterial (PB) and Probable Viral (PV) groups (Table 1) were analyzed in combination with the DB and DV groups, enterovirus and coronavirus 229 E were more common in the combined DV/PV group and rhinovirus was found more commonly in the DB/PB combined group (OR: 1.36, 95% CI: 1.14–1.61) (Figure S1).

#### Blood viruses

Enterovirus and EBV were more common in the DV cases, and HHV6b was more common in DB (OR 1.24, 95% CI 1.00–1.53) (Fig. 2B). When the PB and PV cases were included, only enterovirus was more common in the DV/PV combined group (OR: 0.34, 95% CI: 0.21–0.50) (Figure S1).

#### Blood bacteria

*N. meningitidis* (OR: 3.37, 95% CI: 1.92–5.99), *S. pneumoniae* (OR: 3.89, 95% CI: 2.07–7.59), Group A streptococcus (OR 2.73, 95% CI 1.13–6.09) and *E. coli* (OR 2.7, 95% CI 1.02–6.71) were more commonly detected in DB than DV cases (Fig. 2B). When DB and PB cases were considered together, *Enterococcus faecium* (OR: 1.95, 95% CI: 1.00–3.52) and *Serratia marcescens* (OR: 1.83, 95% CI: 1.01–3.09) were also found more commonly in the bacterial cases.

### CMT in infants vs older children

For most pathogens there were insufficient positive detections in children under 1 year of age to reveal any trends in pathogen detection in cases vs controls (Figure S2) or patients in definite bacterial vs definite viral groups (Figure S3). However, there were some differences between infants and older children.

Enterovirus was significantly more likely to be detected in cases vs controls and patients with definite viral vs bacterial infection in infants, but this was not the case in older children. On further investigation, 24/51 (47%) of infants with enterovirus detected by CMT in blood were diagnosed as having an enterovirus meningitis, with enterovirus detected in CSF as part of the local clinical investigations. HHV6b and HHV7 were significantly more likely to be detected in infants with confirmed bacterial infection than those with confirmed viral infection. Although there was a similar trend in older children, the finding was not statistically significant.

### Quantitative CMT

In order to explore whether quantitative load of pathogen sequences improved the distinction between DB and DV groups, we established quantitative counts for the blood viruses. Enterovirus, EBV and HHV6b showed a trend towards increased frequency of detection in cases than controls at higher viral loads (Figure S4), whilst enterovirus, parvovirus and EBV showed this trend in the comparison between DV or DB cases (Figure S5).

### Combination of CMT and local diagnostics

**Fig. 3:**
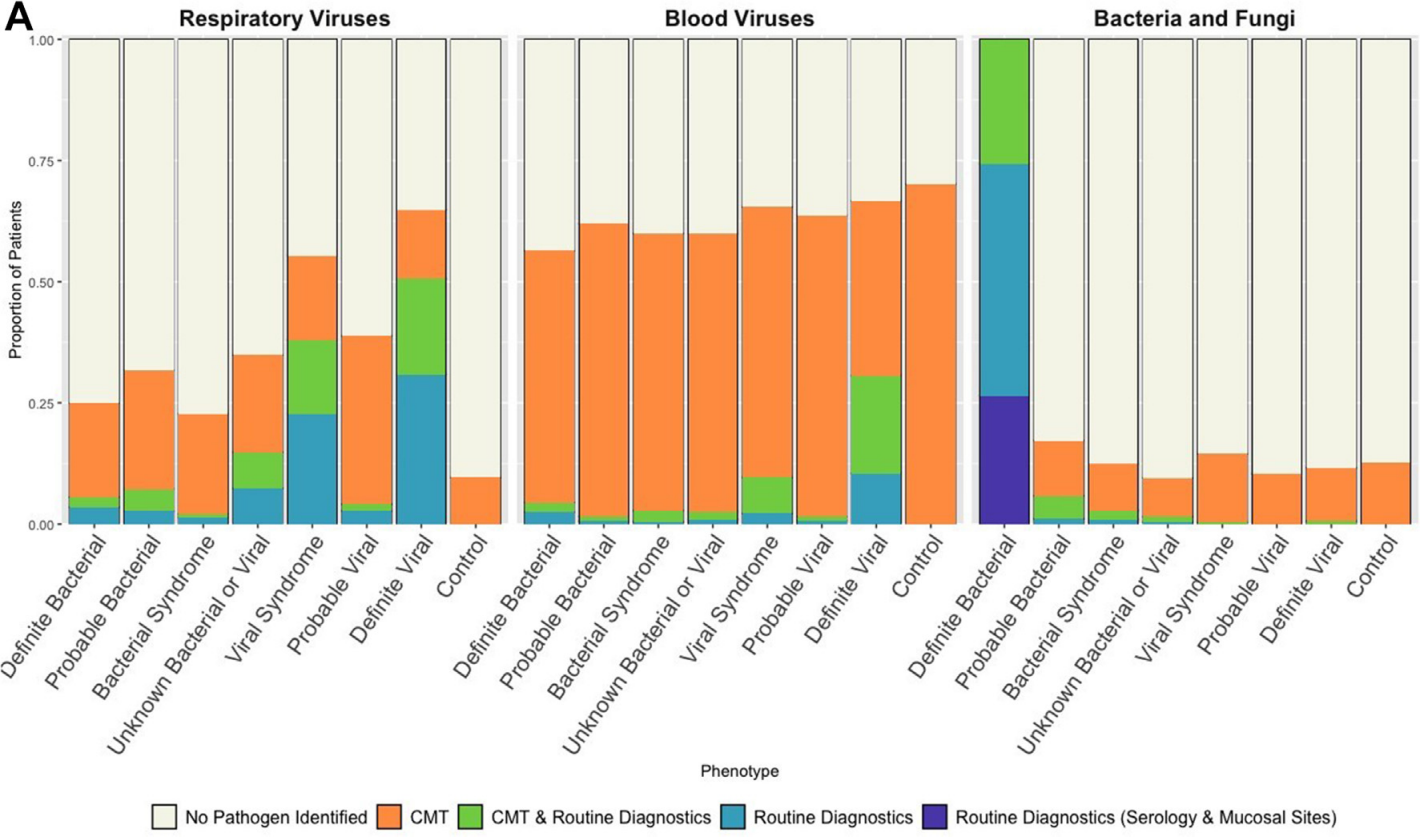

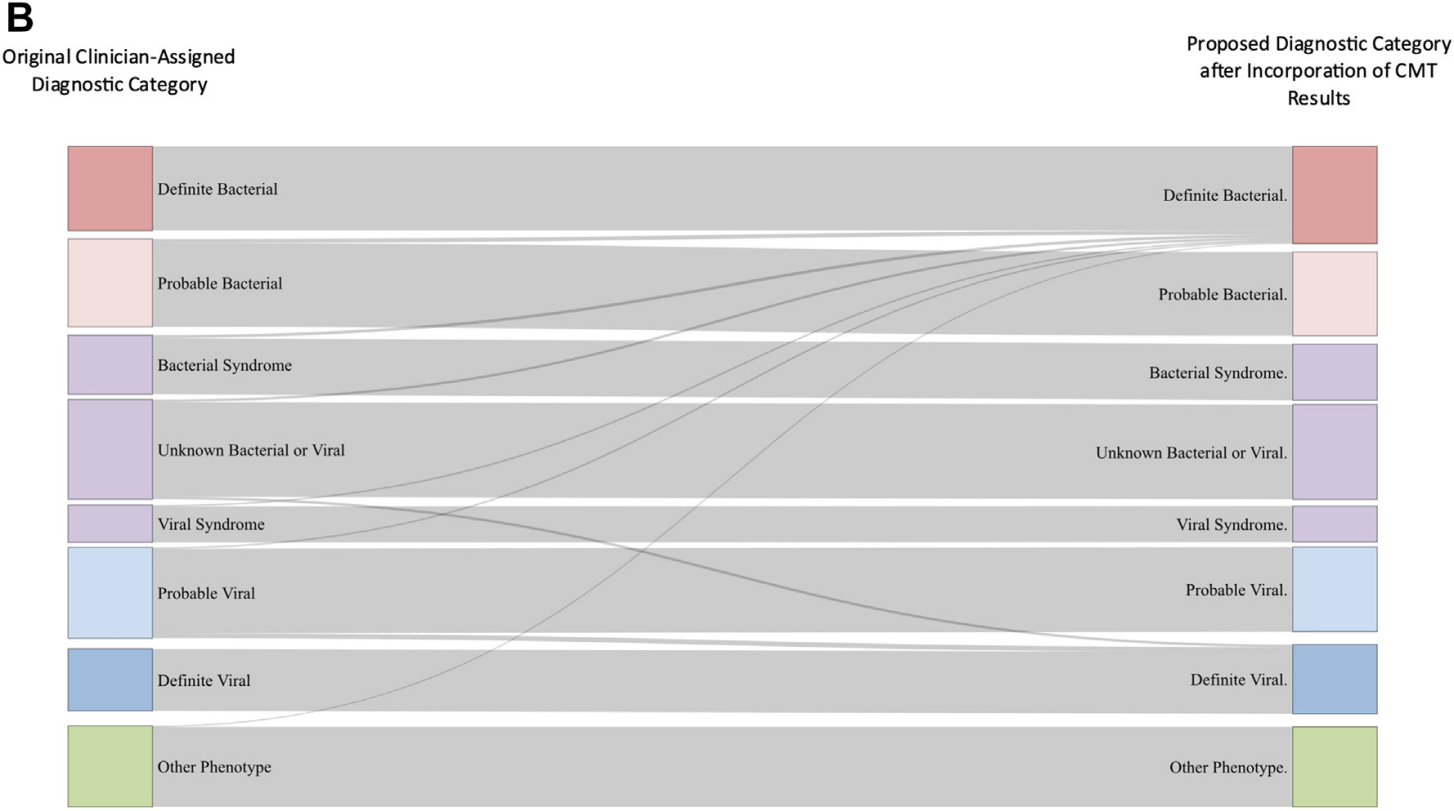
(A) Proportion of patients in each diagnostic category with pathogen detected. Blue bars show pathogens detected by local investigations; green bars show detection by both local and centralized molecular tests (CMT); orange bars show pathogen detection only by CMT. Purple bar shows patients classified as DB on the basis of mucosal detection of specific pathogens (see definitions Table 1). Combined colored bar height indicates proportion of all patients in each diagnostic category with pathogens detected. Individual panels show detection of respiratory viruses (left), blood viruses (middle) and bacterial and fungal pathogens (right). (B) Sankey diagram showing patient reassignment from final diagnostic category based on local investigations alone, to a final diagnostic group based on addition of CMT. The height of each colored bar represents number of patients in each group, and the curves crossing groups indicate patients reassigned based on CMT results. Addition of CMT resulted in an addition of 87 (2%) of patients to the definite bacterial group and 53 (1%) of patients to the definite viral group.

We examined the proportion of patients in each diagnostic category identified by combined local investigations and CMT (Fig. 3A). Overall, 25% of DB and 32% of PB had detected respiratory viruses, and 56% and 62% of DB and PB had detected blood viruses (Fig. 3A). The detection of a virus in blood or throat swab had predictive values of 0.64 and 0.68 respectively for excluding a diagnostic category DB or PB. The detection of some viruses, such as influenza A (0.82), influenza B (0.85), RSV (0.89) and Enterovirus (0.84) on throat swabs, as well as Enterovirus (0.85) in blood samples showed superior predictive values in excluding a DB or PB diagnostic category (Table S11).

In order to evaluate how the CMT results might change assignment to DB or DV or other groups, we reassigned patients from the uncertain categories (unknown bacterial or viral, PB, PV, viral syndrome or bacterial syndrome) based on the CMT results only for pathogens showing significant odds ratios for distinguishing bacterial and viral infection (*N. meningitidis*, *S. pneumoniae*, group A streptococcus, *E. coli* and *M. pneumoniae* for bacteria; Influenza A and B, RSV for respiratory viruses; and EBV and enterovirus for blood viruses). Only 87 patients (2%) were reassigned as DB, and 53 (1%) as DV (Fig. 3B).

## Discussion

Our multi-country prospective observational study aimed to establish the microbiological etiology in febrile children presenting to hospitals in the UK and 9 EU countries. Despite vigorous application of conventional diagnostic approaches, supplemented by molecular methods to detect bacteria and viruses in blood and respiratory secretions, most patients could not be definitively assigned either a bacterial or a viral etiology. The reasons for this are multifactorial and include poor sensitivity of some diagnostic techniques, inability to sample from the site of infection or suboptimal specimen collection. Based on interpretation of available clinical and diagnostic data by local recruiting teams, only 14% had a microbiologically confirmed bacterial infection, and a further 11% had a viral infection with no evidence of bacterial infection. The remaining 75% had differing levels of certainty as to whether bacteria or viruses were the cause of illness. Incorporation of CMT results into the process of assigning patients to a diagnostic category increased the assignment as DB to 16%, and as DV to 12%. Although CMT greatly increased the number of patients with detected viruses, they were detected in all diagnostic categories suggesting that a high proportion of patients with proven or probable bacterial infections were co-infected with viruses. Our inability to clearly distinguish patients with bacterial from viral infection, even after detailed investigation in a prospective research study, was reflected in the clinical management of the patients. A high proportion of all patients in the study (80%), including those with confirmed (71%) or probable (48%) viral infections, were prescribed antibiotics by the medical teams responsible for their care.

Viruses were detected in respiratory samples more commonly in febrile children than in controls overall, but influenza, RSV, enterovirus, and coronavirus OC43 were the only viruses with significantly greater odds of detection in the DV than DB group. Enterovirus and EBV were the only blood viruses more common in children with DV than DB. Therefore, virus detection in blood or respiratory tract had a poor predictive value for excluding bacterial infection.

For bacterial and fungal pathogens, CMT found *N. meningitidis*, *S. pneumoniae*, Group A streptococcus and *E. coli* in blood, and *M. pneumoniae* in throat swabs more commonly in the DB than the DV group. For all other bacteria and fungi, no clear distinction was seen between the rate of detection of bacterial DNA in DB and DV or between cases and controls.

Of note, CMT showed HHV6b and HHV7 were more common in controls than in cases, even though the controls had no features suggestive of infection. As herpes viruses such as EBV, CMV, HHV6 and HHV7 establish persistent infection in healthy individuals,^32^, ^33^, ^34^ detection of these viruses may represent reactivation and not indicate that they are involved in a current illness. We explored whether viral load differed in children with DV and DB. Higher viral load was associated with a higher likelihood of a viral diagnosis for EBV and HHV6b, but not HHV7 or CMV. We speculate that higher rates of detection of blood viruses in controls than cases might reflect suppression of persistent viruses by immune mediators released in response to other infections. This hypothesis will need exploration in future studies.

Our inability to clearly distinguish bacterial from viral infections even with extensive molecular pathogen detection approaches has several possible explanations. Many common bacterially mediated infections such as pneumonia, abdominal or bone and soft tissue infection remain localized, and are associated with bacteremia only in severe cases. Molecular detection of bacterial DNA in blood may be no more sensitive than culture in these cases, and this may reflect the restriction for organism detection of the small (200 μl) volume of blood available for molecular testing. Many of the bacterial and fungal targets detected were found equally in cases and controls, and in DB and DV, and their clinical significance is therefore unclear. In contrast to the difficulty in detecting bacteria in blood, viruses are readily detected using molecular methods on mucosal surfaces or blood. In addition to children with typical viral infections, viruses were detected in a high proportion with proven or probable bacterial infections.

We found a high proportion of patients with confirmed bacterial infections also had viruses identified in blood or throat swabs. Our findings confirm the difficulties in decisions relating to commencing or discontinuing antibiotics in children with confirmed viral infections. As we found respiratory viruses more commonly in febrile patients than in non-febrile controls, it is likely that viral and bacterial infection are not independent, in line with the increasing evidence that viral infections set in motion pathophysiological processes that may increase susceptibility to bacterial infection.

Respiratory viruses damage and inflame the airway mucosa, reduce mucociliary clearance, facilitate bacterial adherence and allow migration or invasion of the airway microbiome into deeper tissue.^35^, ^36^, ^37^, ^38^ Epidemiological studies have shown that peaks of bacterial pneumonia and invasive bacterial infection follow seasonal and pandemic upsurges of influenza,^39^, ^40^, ^41^, ^42^, ^43^, ^44^ with bacterial coinfection implicated in severe influenza-associated disease and death.^35^^,^^45^ Similarly, RSV seasonality has been linked to meningococcal disease,^46^ pneumococcal pneumonia^47^, ^48^, ^49^, ^50^ and bacteraemia.^51^ Studies suggest pneumococcal vaccine reduces influenza-associated hospitalisations^52^, ^53^, ^54^, ^55^, ^56^ and severity of other viral respiratory infections,^57^ and influenza vaccine has been reported to reduce invasive bacterial infections.^58^^,^^59^ *In vitro* studies show a wide range of immunological impairments following viral infection, including impaired pathogen recognition and decreased phagocytosis.^60^^,^^61^ In murine models, influenza coinfection is associated with increased pneumococcal and staphylococcal bacterial loads and disease.^58^^,^^62^, ^63^, ^64^ Thus, the finding that a large proportion of patients in our study with viruses detected also had evidence of bacterial infection supports the view that both viruses and bacteria are contributing to the disease process.

Several previous studies have attempted to determine the etiology of fever or pneumonia in children, including studies in low and middle-income countries.^65^, ^66^, ^67^, ^68^, ^69^ The PERCH,^70^ Drakenstein^66^ and GABRIEL^68^ pneumonia studies attempted to define the role of bacterial and viral pathogens in childhood pneumonia, and D'Acremont et al. explored etiology of outpatient febrile illness in Tanzania.^65^ These studies highlighted the high proportion of febrile episodes associated with viral infection, and also noted that many cases had multiple pathogens detected. Our study combines a detailed molecular pathogen approach with a detailed patient-by-patient clinical categorization performed independently of the CMT data, and enables comparison of the performance of current clinical and research pathogen detection approaches in diagnosing or excluding bacterial infection.

Our study has a number of limitations. The non-febrile control patients were not matched for age, sex, time of year and country, but were an opportunistic cohort of hospitalized children admitted for conditions which did not appear to have an underlying infectious basis. In order to best match the febrile patients, we included a broad range of noninfectious hospital admissions, including orthopedic conditions, and intensive care admissions for surgical, cardiac, or traumatic illnesses, and non-febrile children with cancer undergoing immunosuppressive treatments. They may not be truly representative of the spectrum of viruses and pathogens carried by healthy children in the community. Secondly, our approach applied all CMT assays to all patients, unlike clinical practice in which pathogen tests are selected on a individual patient basis, based on judgement of the likely cause of illness. Whilst our comprehensive approach enabled us to look at pathogen burden in patients, including an examination of pathogens not typically associated with the presenting illness, it did not measure the diagnostic utility of these assays when they are used in individual patients. Despite the large number of patients in bacterial and viral groups, some pathogens that rarely cause disease have a small number of positive detections in our cohort.

Our study shows that the efforts to improve clinical diagnosis by molecular pathogen detection is not adequate to resolve the clinical question of which febrile patients need antibiotics to treat potentially life-threatening bacterial infections, and when antibiotics can be safely withheld. A high proportion of all febrile children evaluated in hospital cannot be conclusively defined as having either a bacterial or viral infection, even when molecular tests supplement conventional culture approaches. Viruses are detected in a high proportion of patients with bacterial infections, and the clinical value of individual pathogen detection in determining treatment is low. New approaches are needed to help clinicians determine which febrile children require antibiotics.

## Contributors

**Writing Group**: Priyen Shah, Marie Voice, Leonides Calvo-Bado, Irene Rivero Calle, Sophie Morris, Ruud Nijman, Claire Broderick, Tisham De, Irini Eleftheriou, Rachel Galassini, Aakash Khanijau, Laura Kolberg, Mojca Kolnik, Aleksandra Rudzate, Manfred Sagmeister, Nina Schweintzger, Fatou Secka, Clare Thakker, Fabian van der Velden, Clementien Vermont, Katarina Vincek, Philipp KA Agyeman, Aubrey Cunnington, Ronald De Groot, Marieke Emonts, Katy Fidler, Rachel Galassini, Taco Kuijpers, Marine Mommert-Tripon, Karen Brengel-Pesce, Francois Mallet, Henriette Moll, Stéphane Paulus, Marko Pokorn, Andrew Pollard, Luregn J Schlapbach, Ching-Fen Shen, Maria Tsolia, Effua Usuf, Michiel van der Flier, Ulrich von Both, Shunmay Yeung, Dace Zavadska, Werner Zenz, Victoria Wright, Enitan D Carrol, Myrsini Kaforou, Federico Martinon-Torres, Colin Fink, Michael Levin, Jethro Herberg.

**Data Curation and Analysis Group**: Priyen Shah, Marie Voice, Myrsini Kaforou, Mike Levin and Jethro Herberg. The data curation and analysis group had access to all the data, vouch for completeness and accuracy of the data presented, and took responsibility for the decision to submit.

## Data sharing statement

Clinical phenotype and CMT data from this study are held by the data management team, and can be made available to other research groups, after approval of a proposal and with a signed data access agreement, after study close-out, or earlier with Consortium agreement. Please contact the corresponding author with an outline of the intended use.

## Declaration of interests

AC received research funding from UK-NIHR and EPSRC. He has unpaid roles at ESPID, and the Excellence in Paediatrics Institute. He filed a patent for a new diagnostic method in children. AP received grant funding from Gates Foundation, Wellcome Trust, Cepi, UK-MRC and NIHR. He received consulting fees from Shionogi. He leads the UK Joint Committee on Vaccination and Immunisation, and was a memeber of WHO-SAGE until 2022. Oxford University has entered into a partnership with AZ for development of COVID19 vaccines. CB received UK-NIHR research funding. FMT received funding from Consorcio Centro de Investigación Biomédica en Red de Enfermedades Respiratorias, Grupos de Referencia Competitiva, and Instituto de Salud Carlos III. MT has unpaid role at the National Committee on Immunization Practices, and at the Scientific Advisory Group of Experts for COVID-19. TK has unpaid roles at the National Working Party on Immunodeficiencies, and the National Advisory Committee on SARS-CoV-2 vaccination. UVB received funding from TeleKasper—Innovationsfonds, German G-BA, and has received funds for lectures at MSD—Workshop Pädiatrie. The other authors have no conflict of interests.
