# Supplementary material for: Relationship between molecular pathogen detection and clinical disease in febrile children across Europe: a multicentre, prospective observational study
Source: Lancet Reg Health Eur. 2023 Jul 26;32:100682. doi: 10.1016/j.lanepe.2023.100682 (PMC10405323; doi:10.1016/j.lanepe.2023.100682)
Supplement: Supplementary Methods [file mmc1.pdf]

Relationship between molecular pathogen detection and clinical disease in febrile children across Europe

Supplementary appendix

Contents

Relationship between molecular pathogen detection and clinical disease in febrile children across Europe 1
Supplementary appendix 1
Contents 1
Membership of the writing group 3
Analysis group. 3
Membership of the PERFORM consortium (https://www.perform2020.org/) 3
PARTNER: Imperial College (Coordinating Centre) (UK) 3
PARTNER: SERGAS (Spain) 4
PARTNER: Riga Stradins University (Latvia) 4
PARTNER: Medical Research Council Unit The Gambia (MRCG) at LSHTM 4
PARTNER: ERASMUS MC-Sophia Children's Hospital (Netherlands) 5
PARTNER: Swiss Pediatric Sepsis Study (Switzerland) 5
PARTNER: University Of Liverpool (UK) 5
PARTNER: National and Kapodistrian University of Athens (Greece)[1] 6
PARTNER: Micropathology Ltd (UK)[1] 6
PARTNER: Medical University of Graz (Austria) 6
PARTNER: London School of Hygiene and Tropical Medicine (UK) 7
PARTNER: Radboud University Medical Center (The Netherlands) 7
PARTNER: Oxford University (UK) 7
PARTNER: Newcastle University, Newcastle Upon Tyne (UK) 8
PARTNER: LMU Munich (Germany) 9
PARTNER: BioMérieux (France) 9
PARTNER: Department of Infectious Diseases, University Medical Centre Ljubljana (Slovenia) 9
PARTNER: Academic Medical Hospital & Sanquin Research Institute, Amsterdam (NL) 9
Supplementary Methods 11
PERFORM handbook 11
Centralized molecular testing methodology 11
Sample preparation for retrospective diagnostic testing 11

Nucleic acid extraction 11
Respiratory pathogen screening 11
Cycling conditions for Rhinovirus/Enterovirus differentiation PCR. 12
Molecular screening for blood pathogens.. 12
qPCR Reaction Conditions 12
PCR cycling conditions for blood pathogen RNA/DNA detection. 13
Quantitative viral qPCR Assays 13
Bacterial and Fungal qPCR Assays 13
Strobe checklist for observationalstudies.. 13
Supplementary Tables 17
Table S1: Participating European clinical recruitment sites in PERFORM.................... 17
Table S2: Targets included in pathogen screening of throat swabs, in centralized molecular tests 17
Table S3: PERFORM viral blood qPCR assays. 18
Table S4: PERFORM bacterial and fungal qPCR assays. 19
Table S5: Investigation of potential confounders.... 21
Table S6: Bacterial detections by clinically indicated pathogen tests in DB patients. 22
Table S7: Viral detections by local, clinically indicated pathogen tests by diagnostic category. 23
Table S8: Frequency of pathogen detection in throat swabs by centralized molecular tests in relation to the clinically-assigned diagnostic category. 24
Table S9: Frequency of virus detection in blood by centralized molecular tests in relation to the clinically-assigned diagnostic category, ... 25
Table S10: Frequency of bacterial/fungal detection in blood by centralized molecular tests in relation to the clinically-assigned diagnostic category 26
Table S11: Predictive values of viruses for ruling in or out a DB or PB infection 27
Supplementary Figures 28
Figure S1: Odds ratios for molecular pathogen identifications in combined Definite and Probable Bacterial groups compared to Definite and Probable Viral groups................. 28
Figure S2: Quantitative molecular detection by increasing viral load quartile – blood viruses in relation to cases vs controls 31
Figure S3: Quantitative molecular detection by increasing viral load quartile – blood viruses in relation to Definite Viral vs Definite Bacterial diagnostic categories. 32

Membership of the writing group

Priyen Shah, Marie Voice, Leonides Calvo-Bado, Irene Rivero Calle, Sophie Morris, Ruud Nijman, Claire Broderick, Tisham De, Irini Eleftheriou, Rachel Galassini, Aakash Khanijau, Laura Kolberg, Mojca Kolnik, Aleksandra Rudzate, Manfred Sagmeister, Nina Schweintzger, Fatou Secka, Clare Thakker, Fabian van der Velden, Clementien Vermont, Katarina Vincek, Philipp KA Agyeman, Aubrey Cunnington, Ronald De Groot, Marieke Emonts, Katy Fidler, Rachel Galassini, Taco Kuijpers, Marine Mommert-Tripon, Karen Brengel-Pesce, Francois Mallet, Henriette Moll, Stéphane Paulus, Marko Pokorn, Andrew Pollard, Luregn J Schlapbach, Ching-Fen Shen, Maria Tsolia, Effua Usuf, Michiel van der Flier, Ulrich von Both, Shunmay Yeung, Dace Zavadska, Werner Zenz, Victoria Wright, Enitan D Carrol, Myrsini Kaforou, Federico Martinon-Torres, Colin Fink, Michael Levin§, Jethro Herberg

§ Corresponding author

Analysis group.

PS, MV, MK, ML and JH had access to all the data, and vouch for completeness and accuracy of the data presented.

Membership of the PERFORM consortium (https://www.perform2020.org/)

PARTNER: Imperial College (Coordinating Centre) (UK)

Chief investigator/PERFORM coordinator:

Michael Levin

Principal and co-investigators; work package leads (alphabetical order)[1]

Aubrey Cunnington (grant application, clinical translation); Tisham De (clinical database); Jethro Herberg (Principal Investigator, grant application); Myrsini Kaforou (grant application, bioinformatics); Victoria Wright (grant application, Scientific Manager)

Research Group (alphabetical order)[1]

Lucas Baumard; Evangelos Bellos; Lachlan Coin, Giselle D'Souza; Rachel Galassini; Dominic Habgood-Coote; Shea Hamilton; Clive Hoggart; Sara Hourmat; Heather Jackson; Naomi Lin; Stephanie Menikou; Samuel Nichols; Ruud Nijman; Ivonne Pena Paz; Oliver Powell, Priyen Shah; Ortensia Vito; Clare Wilson

Clinical recruitment at Imperial College Healthcare NHS Trust, London (alphabetical order)[2]

Amina Abdulla; Ladan Ali; Sarah Darnell; Rikke Jorgensen; Ian Maconochie[3]; Sobia Mustafa; Salina Persand; Ben Walsh

Department of Materials[4]

Molly Stevens (co-investigator); Nayoung Kim, Eunjung Kim, Benjamin Pierce

Clinical recruitment at Brighton and Sussex University Hospitals[5]

Katy Fidler (Principal Investigator); Julia Dudley (Clinical Research Registrar); Research nurses: Vivien Richmond, Emma Tavliavini

[1]Section of Paediatric Infectious Disease, Wright-Fleming Institute, Norfolk Place, London W2 1PG, UK
[2]Children's Clinical Research Unit, St Mary's Hospital, Praed Street, London W2 1NY, UK
[3]Paediatric Emergency Dept, St Mary's Hospital, Praed Street, London W2 1NY, UK
[4]Department of Materials, Royal School of Mines, Prince Consort Rd, London SW7 2AZ, UK
[5]Royal Alexandra Children's Hospital, Brighton, BN2 5BE, UK.

Clinical recruitment at National Cheng Kung University Hospital, Tainan, Taiwan

Ching-Fen Shen (Principal Investigator)[1,2]; Ching-Chuan Liu, Shih-Min Wang (Co-investigators)

[1] Department of Paediatrics, National Cheng Kung University Hospital, College of Medicine, National Cheng Kung University, Tainan, Taiwan;
[2] Institute of Clinical Medicine, College of Medicine, National Cheng Kung University, Tainan, Taiwan

PARTNER: SERGAS (Spain)

Principal Investigators

Federico Martinón-Torres[1], Antonio Salas[1,2]

<u>*GENVIP RESEARCH GROUP*</u> (in alphabetical order):

Fernando Álvez González[1], Cristina Balo Farto[1], Ruth Barral-Arca[1,2], María Barreiro Castro[1], Xabier Bello[1,2], Mirian Ben García[1], Sandra Carnota[1], Miriam Cebey-López[1], María José Curras-Tuala[1,2], Carlos Durán Suárez[1], Luisa García Vicente[1], Alberto Gómez-Carballa[1,2], Jose Gómez Rial[1], Pilar Leboráns Iglesias[1], Federico Martinón-Torres[1], Nazareth Martinón-Torres[1], José María Martinón Sánchez[1], Belén Mosquera Pérez[1], Jacobo Pardo-Seco[1,2], Lidia Piñeiro Rodríguez[1], Sara Pischedda[1,2], Sara Rey Vázquez[1], Irene Rivero Calle[1], Carmen Rodríguez-Tenreiro[1], Lorenzo Redondo-Collazo[1], Miguel Sadiki Ora[1], Antonio Salas[1,2], Sonia Serén Fernández[1], Cristina Serén Trasorras[1], Marisol Vilas Iglesias[1].

[1] Translational Pediatrics and Infectious Diseases, Pediatrics Department, Hospital Clínico Universitario de Santiago, Santiago de Compostela, Spain, and GENVIP Research Group (www.genvip.org), Instituto de Investigación Sanitaria de Santiago, Universidad de Santiago de Compostela, Galicia, Spain.
[2] Unidade de Xenética, Departamento de Anatomía Patolóxica e Ciencias Forenses, Instituto de Ciencias Forenses, Facultade de Medicina, Universidade de Santiago de Compostela, and GenPop Research Group, Instituto de Investigaciones Sanitarias (IDIS), Hospital Clínico Universitario de Santiago, Galicia, Spain
[3] Fundación Pública Galega de Medicina Xenómica, Servizo Galego de Saúde (SERGAS), Instituto de Investigaciones Sanitarias (IDIS), and Grupo de Medicina Xenómica, Centro de Investigación Biomédica en Red de Enfermedades Raras (CIBERER), Universidade de Santiago de Compostela (USC), Santiago de Compostela, Spain

PARTNER: Riga Stradins University (Latvia)

Principal Investigator

Dace Zavadska[1,2]

Other RSU group authors (in alphabetical order):

Anda Balode[1,2], Arta Bārzdiņa[1,2], Dārta Deksne[1,2], Dace Gardovska[1,2], Dagne Grāvele[2], Ilze Grope[1,2], Anija Meiere[1,2], Ieva Nokalna[1,2], Jana Pavāre[1,2], Zanda Pučuka[1,2], Katrīna Selecka[1,2], Aleksandra Rudzate[1,2], Dace Svile[2], Urzula Nora Urbāne[1,2].

[1] Riga Stradins university, Riga, Latvia.
[2] Children clinical university hospital, Riga, Latvia.

PARTNER: Medical Research Council Unit The Gambia (MRCG) at LSHTM

Medical Research Council Unit The Gambia at LSHTM, P O Box 273, Fajara, The Gambia

Principal Investigator

Effua Usuf

Additional Investigators

Kalifa Bojang, Syed M. A. Zaman, Fatou Secka, Suzanne Anderson, Anna Roca, Isatou Sarr, Momodou Saidykhan, Saffiatou Darboe, Samba Ceesay, Umberto D'alessandro

PARTNER: ERASMUS MC-Sophia Children's Hospital (Netherlands)

Principal Investigator

Henriëtte A. Moll[1], Clementien L. Vermont[2]

Research group

Dorine M. Borensztajn[1], Nienke N. Hagedoorn, Chantal Tan [1], Joany Zachariasse [1]

Additional investigator

W Dik [3]

[1] Erasmus MC-Sophia Children's Hospital, Department of General Paediatrics, Rotterdam, the Netherlands
[2] Erasmus MC-Sophia Children's Hospital, Department of Paediatric Infectious Diseases & Immunology, Rotterdam, the Netherlands
[3] Erasmus MC, Department of immunology, Rotterdam, the Netherlands

PARTNER: Swiss Pediatric Sepsis Study (Switzerland)

Principal Investigators:

Philipp KA Agyeman, MD [1], Luregn J Schlapbach, [2,3] Christoph Aebi[1], Christoph Berger[1, 13]

Clinical recruitment at University Children's Hospital Bern for PERFORM:

Christoph Aebi [1], Verena Wyss [1], Mariama Usman [1]

Principal and co-investigators for the Swiss Pediatric Sepsis Study:

Philipp KA Agyeman, MD [1], Luregn J Schlapbach, MD, PhD, FCICM [2,3], Eric Giannoni, MD [4,5], Martin Stocker, MD [6], Klara M Posfay-Barbe, MD [7], Ulrich Heininger, MD [8], Sara Bernhard-Stirnemann, MD [9], Anita Niederer-Loher, MD [10], Christian Kahlert, MD [10], Giancarlo Natalucci, MD [11], Christa Relly, MD [12], Thomas Riedel, MD [13], Christoph Aebi, MD [1], Christoph Berger, MD [12] for the Swiss Pediatric Sepsis Study

[1] Department of Pediatrics, Inselspital, Bern University Hospital, University of Bern, Switzerland
[2] Neonatal and Pediatric Intensive Care Unit, Children's Research Center, University Children's Hospital Zurich, University of Zurich, Zurich, Switzerland
[3]Child Health Research Centre, University of Queensland, and Queensland Children`s Hospital, Brisbane, Australia
[4] Clinic of Neonatology, Department Mother-Woman-Child, Lausanne University Hospital and University of Lausanne, Switzerland
[5] Infectious Diseases Service, Department of Medicine, Lausanne University Hospital and University of Lausanne, Switzerland
[6] Department of Pediatrics, Children's Hospital Lucerne, Lucerne, Switzerland
[7] Pediatric Infectious Diseases Unit, Children's Hospital of Geneva, University Hospitals of Geneva, Geneva, Switzerland
[8] Infectious Diseases and Vaccinology, University of Basel Children's Hospital, Basel, Switzerland
[9] Children's Hospital Aarau, Aarau, Switzerland
[10] Division of Infectious Diseases and Hospital Epidemiology, Children's Hospital of Eastern Switzerland St. Gallen, St. Gallen, Switzerland
[11] Department of Neonatology, University Hospital Zurich, Zurich, Switzerland
[12] Division of Infectious Diseases and Hospital Epidemiology, and Children's Research Center, University Children's Hospital Zurich, Switzerland
[13] Children's Hospital Chur, Chur, Switzerland

PARTNER: University Of Liverpool (UK)

Principal Investigators

Enitan D Carrol[1,2,3], Stéphane Paulus [1,]

Research Group (in alphabetical order):

Elizabeth Cocklin[1], Rebecca Jennings[4], Joanne Johnston[4], Aakash Khanijau[1,] Simon Leigh[1], Karen Newall[4], Sam Romaine[1],

[1]Department of Clinical Infection, Microbiology and Immunology, University of Liverpool Institute of Infection, Veterinary and Ecological Sciences , Liverpool, England
[2]Alder Hey Children's Hospital, Department of Infectious Diseases, Eaton Road, Liverpool, L12 2AP
[3]Liverpool Health Partners, 1st Floor, Liverpool Science Park, 131 Mount Pleasant, Liverpool, L3 5TF
[4]Alder Hey Children's Hospital, Clinical Research Business Unit, Eaton Road, Liverpool, L12 2AP

PARTNER: National and Kapodistrian University of Athens (Greece)[1]

Principal investigator:

Professor Maria Tsolia (all activities)

Investigator/Research fellow:

Irini Eleftheriou (all activities)

Additional investigators:

Maria Tambouratzi (Recruitment); Antonis Marmarinos (Lab, Quality Manager); Marietta Xagorari (Lab); Kelly Syggelou; Nikos Spyridis

[1] 2nd Department of Pediatrics, National and Kapodistrian University of Athens, "P. and A. Kyriakou" Children's Hospital, Thivon and Levadias, Goudi, Athens

PARTNER: Micropathology Ltd (UK)[1]

Principal Investigator:

Professor Colin Fink, Clinical Microbiologist

Additional investigator

Dr Marie Voice, Post doc scientist; Dr. Leo Calvo-Bado, Post doc scientist; Sophie Morris, Miss Jennifer Blackmore, Miss Rebekah Harrison

[1] Micropathology Ltd, The Venture Center, University of Warwick Science Park, Sir William Lyons Road, Coventry, CV4 7EZ.

PARTNER: Medical University of Graz (Austria)

Principal Investigator:

Werner Zenz[1] (all activities)

Co-investigators (in alphabetical order)

Benno Kohlmaier[1] (all activities); Nina A. Schweintzger[1] (all activities); Manfred G. Sagmeister[1] (study design, consortium wide sample management)

Research team

Daniela S. Kohlfürst[1] (study design); Christoph Zurl[1] (BIVA PIC); Alexander Binder[1] (grant application)

Recruitment team, data managers, (in alphabetical order):

Susanne Hösele[1], Manuel Leitner[1], Lena Pölz[1], Glorija Rajic[1],

Clinical recruitment partners (in alphabetical order):

Sebastian Bauchinger[1], Hinrich Baumgart[6], Martin Benesch[3], Astrid Ceolotto[1], Ernst Eber[2], Siegfried Gallistl[1], Gunther Gores[5], Harald Haidl[1], Almuthe Hauer[1], Christa Hude[1], Markus

Keldorfer[5], Larissa Krenn[4], Heidemarie Pilch[5], Andreas Pfleger[2], Klaus Pfurtscheller[4], Gudrun Nordberg[5], Tobias Niedrist[8], Siegfried Rödl[4], Andrea Skrabl-Baumgartner[1], Matthias Sperl[7], Laura Stampfer[5], Volker Strenger[3], Holger Till[6], Andreas Trobisch[5], Sabine Löffler[5]

[1] Department of Pediatrics and Adolescent Medicine, Division of General Pediatrics, Medical University of Graz, Graz, Austria
[2] Department of Pediatric Pulmonology, Medical University of Graz, Graz, Austria
[3] Department of Pediatric Hematooncoloy, Medical University of Graz, Graz, Austria
[4] Paediatric Intensive Care Unit, Medical University of Graz, Graz, Austria
[5] University Clinic of Paediatrics and Adolescent Medicine Graz, Medical University Graz, Graz,Austria
[6] Department of Paediatric and Adolescence Surgery, Medical University Graz, Graz, Austria
[7] Department of Pediatric Orthopedics, Medical University Graz, Graz, Austria
[8] Clinical Institute of Medical and Chemical Laboratory Diagnostics, Medical University Graz, Graz, Austria

PARTNER: London School of Hygiene and Tropical Medicine (UK)

Principal Investigator:

Dr Shunmay Yeung[1,2 3] PhD, MBBS, FRCPCH, MRCP, DTM&H

Research Group:

Dr Juan Emmanuel Dewez[1]; Prof Martin Hibberd [1]; Mr David Bath[2]; Dr Alec Miners[2]; Dr Ruud Nijman[3]; Dr Elizabeth Fitchett, Dr Catherine Wedderburn[1]; Ms Anne Meierford[1]; Dr Baptiste Leurent[4]

[1]Faculty of Infectious and Tropical Disease, London School of Hygiene and Tropical Medicine, London, UK
[2]Faculty of Public Health and Policy, London School of Hygiene and Tropical Medicine, London, UK
[3] Section of Paediatric Infectious Disease, St. Mary's Hospital Imperial College Hospital, London, UK

PARTNER: Radboud University Medical Center (The Netherlands)

Principal Investigators:

Ronald de Groot [1], Michiel van der Flier [1,2,3], Marien I. de Jonge[1]

Co-investigators Radboud University Medical Center (in alphabetical order):

Koen van Aerde[1,2], Wynand Alkema[1], Bryan van den Broek[1], Jolein Gloerich[1], Alain J. van Gool[1], Stefanie Henriet[1,2], Martijn Huijnen[1], Ria Philipsen[1], Esther Willems[1]

Investigators PeDBIG PERFORM DUTCH CLINICAL NETWORK (in alphabetical order):

G.P.J.M. Gerrits[8], M. van Leur[8,] J. Heidema [4],L. de Haan[1,2] C.J. Miedema [5], C. Neeleman [1] C.C. Obihara [6], G.A. Tramper-Stranders7[6]

[1] Radboud Center for Infectious Diseases, Radboudumc, Nijmegen, the Netherlands and Section Pediatric Infectious Diseases, Laboratory of Medical Immunology, Radboud Institute for Molecular Life Sciences
[2] Pediatric Infectious Diseases and Immunology, Amalia Children's Hospital, Nijmegen, The Netherlands
[3] Pediatric Infectious diseases and Immunology, Wilhelmina Children's Hospital, University Medical Center Utrecht, Utrecht, The Netherlands
[4] St. Antonius Hospital, Nieuwegein, The Netherlands
[5] Catharina Hospital, Eindhoven, The Netherlands
[6] ETZ Elisabeth, Tilburg, The Netherlands
[7] Franciscus Gasthuis, Rotterdam, The Netherlands
[8] Canisius Wilhelmina Hospital, Nijmegen, The Netherlands

PARTNER: Oxford University (UK)

Principal Investigators

Andrew J. Pollard[1,2], Rama Kandasamy[1,2], Stéphane Paulus [1,2]

Additional Investigators

Michael J. Carter[1,2], Daniel O'Connor[1,2], Sagida Bibi[1,2], Dominic F. Kelly[1,2], Meeru Gurung[3], Stephen Thorson[3], Imran Ansari[3], David R. Murdoch[4], Shrijana Shrestha[3], Zoe Oliver[5]

[1] Oxford Vaccine Group, Department of Paediatrics, University of Oxford, Oxford, United Kingdom.
[2]NIHR Oxford Biomedical Research Centre, Oxford, United Kingdom.
[3]Paediatric Research Unit, Patan Academy of Health Sciences, Kathmandu, Nepal.
[4]Department of Pathology, University of Otago, Christchurch, New Zealand.
[5] Department of Paediatrics, University of Oxford.

PARTNER: Newcastle University, Newcastle Upon Tyne (UK)

Principal Investigator:

Marieke Emonts [1,2,3] (all activities)

Co-investigators

Emma Lim[2,3,7] (all activities); Lucille Valentine[4]

Recruitment team (alphabetical), data-managers, and GNCH Research unit:

Karen Allen[5], Kathryn Bell[5], Adora Chan[5], Stephen Crulley[5], Kirsty Devine[5], Daniel Fabian[5], Sharon King[5], Paul McAlinden[5], Sam McDonald[5], Anne McDonnell2,[5], Ailsa Pickering[2,5], Evelyn Thomson[5], Amanda Wood[5], Diane Wallia[5], Phil Woodsford[5]

Sample processing:

Frances Baxter[5], Ashley Bell[5], Mathew Rhodes[5]

PICU recruitment

Rachel Agbeko[8]; Christine Mackerness[8];

Students MOFICHE

Bryan Baas[2], Lieke Kloosterhuis[2], Wilma Oosthoek[2]

Students/medical staff PERFORM

Tasnim Arif[6], Joshua Bennet[2], Kalvin Collings[2], Ilona van der Giessen[2], Alex Martin[2], Aqeela Rashid[6], Emily Rowlands[2], Joshua Soon[2], Gabriella de Vries[2], Fabian van der Velden[2]

Engagement work/ethics/cost effectiveness

Lucille Valentine [4], Mike Martin[9], Ravi Mistry[2]

[1] Translational and Clinical Research Institute, Newcastle University, Newcastle upon Tyne UK
[2]Great North Children's Hospital, Paediatric Immunology, Infectious Diseases & Allergy, Newcastle upon Tyne Hospitals NHS Foundation Trust, Newcastle upon Tyne, United Kingdom.
[3]NIHR Newcastle Biomedical Research Centre based at Newcastle upon Tyne Hospitals NHS Trust and Newcastle University, Westgate Rd, Newcastle upon Tyne NE4 5PL, United Kingdom
[4]Newcastle University Business School, Centre for Knowledge, Innovation, Technology and Enterprise (KITE), Newcastle upon Tyne, United Kingdom
[5]Great North Children's Hospital, Research Unit, Newcastle upon Tyne Hospitals NHS Foundation Trust, Newcastle upon Tyne, United Kingdom.
[6]Great North Children's Hospital, Paediatric Oncology, Newcastle upon Tyne Hospitals NHS Foundation Trust, Newcastle upon Tyne, United Kingdom.
[7]Population Health Sciences Institute, Newcastle University, Newcastle upon Tyne, UK
[8]Great North Children's Hospital, Paediatric Intensive Care Unit, Newcastle upon Tyne Hospitals NHS Foundation Trust, Newcastle upon Tyne, United Kingdom.
[9]Northumbria University, Newcastle upon Tyne, United Kingdom.

PARTNER: LMU Munich (Germany)

Principal Investigator:

Ulrich von Both[1,2] MD, FRCPCH (all activities)

Research group:

Laura Kolberg[1] MSc (all activities);

Manuela Zwerenz[1] MSc, Judith Buschbeck[1] PhD

Clinical recruitment partners (in alphabetical order):

Christoph Bidlingmaier[3], Vera Binder[4], Katharina Danhauser[5], Nikolaus Haas[10], Matthias Griese[6], Matthias Kappler[6], Eberhard Lurz[7], Georg Muench[8], Karl Reiter[9], Carola Schoen[9]

[1] Div. Paediatric Infectious Diseases, Dr. von Hauner Children's Hospital, University Hospital, Ludwig-Maximilians University (LMU), Munich, Germany
[2] German Center for Infection Research (DZIF), Partner Site Munich, Munich, Germany
[3]Div. of General Paediatrics, [4]Div. Paediatric Haematology & Oncology, [5]Div. of Paediatric Rheumatology, [6]Div. of Paediatric Pulmonology, [7]Div. of Paediatric Gastroenterology, [8]Neonatal Intensive Care Unit, [9]Paediatric Intensive Care Unit Hauner Children's Hospital, University Hospital, Ludwig Maximilians University (LMU), Munich, Germany, [10]Department Pediatric Cardiology and Pediatric Intensive Care, University Hospital, Ludwig Maximilians University (LMU), Munich, Germany

PARTNER: BioMérieux (France)

Principal Investigator:

François Mallet[1,2, 3], Karen Brengel-Pesce[1,2,3]

Research Group:

Marine Mommert-Tripon[1,2], Audrey Augusto[1,2], Laurence Generenaz[1,2], Guy Oriol[1,2], Sylvie Pons[1,2]

[1] Open Innovation & Partnerships (OIP), bioMérieux S.A., Marcy l'Etoile, France
[2] Joint research unit Hospice Civils de Lyon - bioMérieux, Centre Hospitalier Lyon Sud, 165 Chemin du Grand Revoyet, 69310 Pierre-Bénite, France
[3] EA 7426 Pathophysiology of Injury-induced Immunosuppression, University of Lyon1-Hospices Civils de Lyon-bioMérieux, Hôpital Edouard Herriot, 5 Place d'Arsonval, 69437 Lyon Cedex 3, France

PARTNER: Department of Infectious Diseases, University Medical Centre Ljubljana (Slovenia)

Principal Investigator:

Marko Pokorn[1,2,3] MD, PhD

Research Group:

Mojca Kolnik[1] MD, Katarina Vincek[1] MD, Tina Plankar Srovin[1] MD, PhD, Natalija Bahovec[1] MD, Petra Prunk[1] MD, Veronika Osterman[1] MD, Tanja Avramoska[1] MD

[1] Department of Infectious Diseases, University Medical Centre Ljubljana, Japljeva 2, SI-1525 Ljubljana, Slovenia
[2]University Childrens' Hospital, University Medical Centre Ljubljana, Ljubljana, Slovenia
[3] Department of Infectious Diseases and Epidemiology, Faculty of Medicine, University of Ljubljana, Slovenia

PARTNER: Academic Medical Hospital & Sanquin Research Institute, Amsterdam (NL)

Principal Investigator:

Taco Kuijpers [1,2]

Co-investigators

Ilse Jongerius [2]

Recruitment team:

J.M. van den Berg[1], D. Schonenberg[1], A.M. Barendregt[1], D. Pajkrt[1], M. van der Kuip[1,3], A.M. van Furth[1,3]

Students PERFORM

Evelien Sprenkeler [2], Judith Zandstra [2],

Technical support PERFORM

G. van Mierlo [2], J. Geissler [2]

[1] Amsterdam University Medical Center (Amsterdam UMC), location Academic Medical Center (AMC), Dept of Pediatric Immunology, Rheumatology and Infectious Diseases, University of Amsterdam, Amsterdam, the Netherlands
[2] Sanquin Research Institute, & Landsteiner Laboratory at the AMC, University of Amsterdam, Amsterdam, the Netherlands.
[3] Amsterdam University Medical Center (Amsterdam UMC), location Vrije Universiteit Medical Center (VUMC), Dept of Pediatric Infectious Diseases and Immunology, Free University (VU), Amsterdam, the Netherlands (former affiliation)

Supplementary Methods

PERFORM handbook

The PERFORM handbook includes the internationally-agreed consortium clinical study protocol, the clinical definitions used, and the Case Record Form. It can be downloaded at this link: https://imperialcollegelondon.box.com/shared/static/wutmj84tbqsuzequdb04ae5zwzz3x382.pdf

Centralized molecular testing methodology

Sample preparation for retrospective diagnostic testing

Swabs were thawed from -80ºC to room temperature and vortexed thoroughly. A 200 µL aliquot of eNat® medium was transferred to a 96-well lysis plate for nucleic acid extraction. EDTA whole blood samples were thawed from -80ºC to room temperature and mixed thoroughly. To ensure complete release of DNA from fungal and bacterial cells, a 200 µL aliquot of blood was enzymatically treated by adding 50 µL of pre lysis buffer for 1 hour at 37ºC (20 mM Tris-HCL pH8, 2 mM EDTA pH8, 1.20% Triton X-100, 20 mg/mL Lysozyme, 1 mg/mL Lysostaphin, all supplied by Sigma-Aldridge, UK). An additional 200 µL of blood was taken for virological assessment, but was not pre-treated prior to extraction.

Nucleic acid extraction

Total nucleic acid was extracted from samples using a Maxwell® HT DNA kit customized for Micropathology Ltd (Promega, USA). Samples were extracted in 96-well format using a KingFisher Flex System (ThermoFisher Scientific). Nucleic acid extracts were stored at -20ºC prior to diagnostic testing.

Respiratory pathogen screening

Swab extracts were analysed via the Luminex® NxTAG® Respiratory Pathogen Panel (RPP) kit; a CE-marked IVD. A 35 µL aliquot of extract was applied to each lyophilized test well as per the manufacturer's instructions. First round amplification was performed on an Eppendorf MasterCycler X50S thermal block. The resulting PCR products were analysed using a MAGPIX® instrument with xPONENT® (Version 4.2 Build 1705) and SYNCT® (Version 1.1.181.0) software. Assay performance was monitored by the addition of NATtrol™ Respiratory Pathogen Panel-1 controls to every test run (Zeptometrix®, USA).

The RPP assay kit provides with respect to Picornaviruses a combined output for both Rhinovirus and Enterovirus. Therefore, an additional nested PCR assay was used to differentiate these two viruses in positive samples. Primer sequences were modified from those originally published by C. *Steininger et al, 2001 and S.J Read et al* and targeted the 5' UTR region. A BIOMETRA T300 Thermal block was used for first round amplification and a LightCycler®480 II for secondary amplification and melt analyses (see Table below for cycling conditions).

Cycling conditions for Rhinovirus/Enterovirus differentiation PCR.

1° ROUND - BIOMETRA T300 Thermal block				
Step	TEMP (°C)	TIME (s)	RAMP (°C/s)	Cycles
RT	45	600	4.0	x1
Denaturation	94	120	4.0	x1
PCR	95	20	4.0	x30
	60	20	4.0	
	72	20	4.0	
2° ROUND -LightCycler® 480 II				
Step	TEMP (°C)	TIME (s)	RAMP (°C/s)	Cycles
Denaturation	95	100	4.4	x1
Amplification	95	20	4.4	x27
	55	20	2.2	
	74	20	4.4	
Melt curve	71	1	2.2	x1
	95	Continuous*	0.11	
Cooling	40	1	2.2	x1

*Continuous with 5 Acquisitions/°C. RT: Reverse Transcription

Rhinovirus/Enterovirus positivity was determined by a melt peak (T_m) at approximately 80.5ºC and 84.5ºC, respectively. T_m shifts of ±2ºC were routinely observed due to serotype-specific mutations within the amplicon sequence. The analytical sensitivity of the assay was assessed using Vircell Amplirun® Rhinovirus and Enterovirus RNA controls (Launch Diagnostics, UK). A 95% positive detection rate was determined at 25 and 81 RNA copies/test, respectively. In validation tests no cross-reactivity between the Rhinovirus and Enterovirus primers was observed when testing 27 independently verified picornavirus positive samples using an external quality assurance panel (QMCD, UK).

Molecular screening for blood pathogens

qPCR Reaction Conditions

Hydrolysis probe-based qPCR assays were used to detect 9 viral, 24 bacterial and 6 fungal gene targets from whole blood nucleic acid extracts. For viral assays, 10 µl of nucleic acid template was added to each reaction mix containing 12.5 µl of LightCycler®480 Probes Master (Roche, Switzerland), 2.5 µl of primers @ 10-20 µM and 2.5 µl of probe @ 3-4 µM (IDT, eurofins, Sigma-Aldridge, TIB MOLBIOL). This reaction mix was doubled for bacterial and fungal assays to give a 50 µL total reaction volume. A Roche LightCycler® 480 II was used for single round amplification and analysis. Cycling conditions are shown below.

Table A

RNA AMPLIFICATION (LightCycler® 480 II)				
Step	TEMP (°C)	TIME (s)	RAMP (°C/s)	Cycles
RT	50	300	4.4	x1
Denaturation	95	20	4.4	x1
Amplification & Acquisition	95	20	4.4	x45
	60	45	2.2	
Cooling	40	10	2.2	x1

Table B

DNA AMPLIFICATION (LightCycler® 480 II)				
**Step**	**TEMP (°C)**	**TIME (s)**	**RAMP (°C/s)**	**Cycles**
**Denaturation**	95	420	4.4	x1
**Amplification & Acquisition**	95	10	4.4	x45-50
	56-62	40	2.2	
	72	1	2.2	
**Cooling**	40	10	1.5	x1

PCR cycling conditions for blood pathogen RNA/DNA detection. Showing conditions for viral RNA amplification (A) and viral/bacterial/fungal DNA amplification (B).

Quantitative viral qPCR Assays

Viral primer/probes were designed using IDT PrimerQuest™ software or modified from published sequences. Additional *in silico* analysis and specificity checks were performed using NCBI BLAST Sequence Analysis Tools (Madden T. The BLAST Sequence Analysis Tool. :15). The viral PCR multiplexing strategy, gene targets and assay LLoD (as defined by a 95% probability of true detection) are shown in Table S4. Standard curves were applied to all assays and were calibrated in house using positive reference material quantified by Droplet Digital™ PCR (BIO-RAD, USA). An exception was for Picornavirus family members, where it was not possible to accurately quantify all circulating serotypes. LightCycler®480 II's absolute quantification/fit point analysis was used to attain the cycle threshold (Ct) values and associated viral loads for positive samples. All viral loads were reported in DNA copies/ml, with no values reported below the assay's theoretical limit of detection (100 DNA or RNA copies/ml, equivalent to 1 viral genome/test). All assays demonstrated linearity up to 10^7 viral genomes/mL, with samples exceeding this viral load being titrated until they fell within the linear quantification range.

Bacterial and Fungal qPCR Assays

The PERFORM bacterial and fungal qPCR panels are described in Table S5. All qPCR primers and probes were modified or re-designed by Micropathology Ltd using RealTime qPCR-PrimerQuest™ from IDT. Individual pathogens were detected using highly species-specific primer/probe sets, whereas more conserved gene regions provided a broader coverage for genus targets. For fungal assays, multiple probes were used to detect species-specific regions within a conserved gene. To avoid false positive results from contaminating environmental organisms, positive results with a high Ct (low bacterial/fungal load) were repeated for confirmation (See Table S5 for lower Ct range). Alternatively, Sanger sequencing of the 16s rRNA or 18s rRNA gene was used to confirm or further speciate a qPCR positive result.

STROBE Checklist for Observational Studies

Section of paper	Item No.	Recommendation	Page No.
**Title and abstract**	1	(*a*) Indicate the study's design with a commonly used term in the title or the abstract	1
		(*b*) Provide in the abstract an informative and balanced summary of what was done and what was found	1,2
**Introduction**			
Background/rationale	2	Explain the scientific background and rationale for the investigation being reported	2,3
Objectives	3	State specific objectives, including any prespecified hypotheses	3
**Methods**			
Study design	4	Present key elements of study design early in the paper	3,4
Setting	5	Describe the setting, locations, and relevant dates, including periods of recruitment, exposure, follow-up, and data collection	3,4 & Supp p11
Participants	6	(*a*) *Cohort study*—Give the eligibility criteria, and the sources and methods of selection of participants. Describe methods of follow-up<br>*Case-control study*—Give the eligibility criteria, and the sources and methods of case ascertainment and control selection. Give the rationale for the choice of cases and controls<br>*Cross-sectional study*—Give the eligibility criteria, and the sources and methods of selection of participants	3,4,5 & Supp p11
		(*b*) *Cohort study*—For matched studies, give matching criteria and number of exposed and unexposed<br>*Case-control study*—For matched studies, give matching criteria and the number of controls per case	N/A
Variables	7	Clearly define all outcomes, exposures, predictors, potential confounders, and effect modifiers. Give diagnostic criteria, if applicable	4,5,6

Data sources/ measurement	8*	For each variable of interest, give sources of data and details of methods of assessment (measurement). Describe comparability of assessment methods if there is more than one group	4,5 & Supp p11
Bias	9	Describe any efforts to address potential sources of bias	Supp p11
Study size	10	Explain how the study size was arrived at	5
Quantitative variables	11	Explain how quantitative variables were handled in the analyses. If applicable, describe which groupings were chosen and why	5,8
Statistical methods	12	(*a*) Describe all statistical methods, including those used to control for confounding	5,8
		(*b*) Describe any methods used to examine subgroups and interactions	5,8
		(*c*) Explain how missing data were addressed	N/A
		(*d*) *Cohort study*—If applicable, explain how loss to follow-up was addressed<br>*Case-control study*—If applicable, explain how matching of cases and controls was addressed<br>*Cross-sectional study*—If applicable, describe analytical methods taking account of sampling strategy	N/A
		(*e*) Describe any sensitivity analyses	N/A
**Results**			
Participants	13*	(a) Report numbers of individuals at each stage of study—eg numbers potentially eligible, examined for eligibility, confirmed eligible, included in the study, completing follow-up, and analysed	5
		(b) Give reasons for non-participation at each stage	Fig 1B
		(c) Consider use of a flow diagram	Fig 1B
Descriptive data	14*	(a) Give characteristics of study participants (eg demographic, clinical, social) and information on exposures and potential confounders	Table 1
		(b) Indicate number of participants with missing data for each variable of interest	N/A

		(c) *Cohort study*—Summarise follow-up time (eg, average and total amount)	N/A
Outcome data	15*	*Cohort study*—Report numbers of outcome events or summary measures over time	*7,8,11,12*
		Case-control study—Report numbers in each exposure category, or summary measures of exposure	
		Cross-sectional study—Report numbers of outcome events or summary measures	
Main results	16	(*a*) Give unadjusted estimates and, if applicable, confounder-adjusted estimates and their precision (eg, 95% confidence interval). Make clear which confounders were adjusted for and why they were included	7-13
		(*b*) Report category boundaries when continuous variables were categorized	N/A
		(*c*) If relevant, consider translating estimates of relative risk into absolute risk for a meaningful time period	N/A
Other analyses	17	Report other analyses done—eg analyses of subgroups and interactions, and sensitivity analyses	11-13
**Discussion**			
Key results	18	Summarise key results with reference to study objectives	13-15
Limitations	19	Discuss limitations of the study, taking into account sources of potential bias or imprecision. Discuss both direction and magnitude of any potential bias	14
Interpretation	20	Give a cautious overall interpretation of results considering objectives, limitations, multiplicity of analyses, results from similar studies, and other relevant evidence	13,14
Generalisability	21	Discuss the generalisability (external validity) of the study results	14
Other information			
Funding	22	Give the source of funding and the role of the funders for the present study and, if applicable, for the original study on which the present article is based	8

Supplementary Tables

***Table S1**: Participating European clinical recruitment sites in PERFORM*

Country	Participating site	Ethics Approval Number
Spain	Hospital Clínico Universitario de Santiago de Compostela	2016/331
United Kingdom	St Mary's Hospital, Imperial College Healthcare NHS Trust, London<br>Alder Hey Children's Hospital, Liverpool<br>Great North Children's Hospital, Newcastle-upon-Tyne<br>John Radcliffe Hospital, Oxford<br>Royal Alexandra Children's Hospital, Brighton	16/LO/1684
Netherlands	Sophia's Children's Hospital, Rotterdam<br>Academic Medical Centre, Amsterdam<br>Radboudumc, Nijmegen	NL58103.091.16
Greece	P. and A. Kyriakou Children's Hospital, Athens	415/13.06.16
Austria	Medizinische Universität Graz, Graz	28-518 ex 15/16
Slovenia	University Medical Centre Ljubljana	0120-483/2016-3
Latvia	Children's Clinical University Hospital, Riga	1/16-07-14
Germany	Dr. von Hauner Children's Hospital, Ludwig-Maximilians-University, Munich	699-16
Switzerland	University Children's Hospital, Universität Bern, Bern	2016-01835

***Table S2**: Targets included in pathogen screening of throat swabs, in centralized molecular tests*

**Viral Targets**		
Influenza A	Rhinovirus/Enterovirus	Adenovirus
Influenza A H1	Parainfluenza virus 1	Coronavirus HKU1
Influenza A H3	Parainfluenza virus 2	Coronavirus NL63
Influenza B	Parainfluenza virus 3	Coronavirus 229E
Respiratory Syncytial Virus A	Parainfluenza virus 4	Coronavirus OC43
Respiratory Syncytial Virus B	Human Metapneumovirus	Human Bocavirus
**Bacterial targets**		
Chlamydophila pneumoniae	*Mycoplasmoides pneumoniae*	*Legionella pneumophila*

***Table S3**: PERFORM viral blood qPCR assays.*

Assay	Gene Target	Dye/Detection Filter	95% Detection Threshold (log copies/mL)	Original Sequence Citation
CMV DNA	HXFL4	FAM (465-510)	2.50 (CI 2.36 - 2.81)	*Gunson R.N. et al, 2001*
EBV DNA	BNRF1	Red610 (533-610)	2.79 (CI 2.65-3.02)	*Gunson R.N. et al, 2001*
Av DNA	Hexon	Cy 5 (618-660)	2.54 (CI 2.39-2.84)	*Heim A. et al, 2003*
HHV-6A DNA	U57	FAM (465-510)	2.35 (CI 2.14-2.85)	*Leibovitch E.C. et al, 2014*
HHV-6B DNA	U57	VIC (533-580)	2.06 (CI 2.04-2.35)	*Leibovitch E.C. et al, 2014*
HHV-7 DNA	U42	Cy 5 (618-660)	2.24 (CI 2.07-2.36)	*Micropathology Ltd*
EV RNA	5′NTR	FAM (465-510)	3.13 (CI 2.8-4.17)	*Pabbaraju K.. et al, 2015*
PeV RNA	5′NTR	VIC (533-580)	3.67 (CI 3.46-4.22)	*Pabbaraju K. et al, 2015*
B19 DNA	NS1	FAM (465-510)	2.37 (CI 2.22-2.65)	*Micropathology Ltd*

4 multiplex hydrolysis probe-based assays were used to detect 9 viral genes. Primer/probe sequences were designed at Micropathology Ltd or represented modified versions of those cited. Av: Adenovirus, B19; Parvovirus B19, CMV; Cytomegalovirus, EBV; Epstein-Barr virus, Ev; Enterovirus, HHV; Human Herpes virus, PeV; Parechovirus.

Table S4: *PERFORM bacterial and fungal qPCR assays.*

Organism (bacteria)	Gene	Dye/Detection Filter	LLoD (Ct range)	Original Sequence Citation	Comments
Neisseria meningitidis	ctrA	FAM (465-510)	35.8-38.9	*Naoko C.et al, 2009/WHO*	
Haemophilus influenzae	hpd	HEX (533-580)	35.0-37.49	*Wang X. et al, 2011*	
Streptococcus pneumoniae #1	lytA	Cy 5 (618-660)	36.24-38.94	*Carvalho Mda G. et al, 2007*	*One of two S. pneumoniae specific gene targets.*
Group A Streptococcus	dnaseB	FAM (465-510)	34.50-35.71	*Micropathology Ltd*	
Group B Streptococcus	hemolysin	HEX (533-580)	32.88-36.42	*de Zoysa A. et al, 2012*	
Group A, B, G Streptococcus	scpB gene	Cy 5 (618-660)	34.20-37.5	*Micropathology Ltd*	*Group A & Group B confirmation. Also detects Group G.*
Streptococcus genus #1	tuf	FAM (465-510)	28.36-33.11	*Micropathology Ltd*	*Primary gene target covering viridans Streptococcus spp.*
Streptococcus genus #2	16SrRNA	HEX (533-580)	26.02-29.86		*Secondary gene target covering viridans Streptococcus spp.*
Streptococcus pneumoniae #2	lytA	Cy 5 (618-660)	36.24-38.94	*Carvalho Mda G. et al, 2007*	*One of two S. pneumoniae specific gene targets.*
Staphylococcus aureus	spa	FAM (465-510)	37.59-39.85	*Okolie C. et al, 2015*	
Staph spp.	tuf	HEX (533-580)	32.33-35.12	*Hwang S.M et al, 2011*	*Provides coverage of coagulase negative species*
16S rRNA	16S rRNA	Cy 5 (618-660)	29.8-30.74	*Clifford R.J et al, 2012*	*Detects pan bacterial 16S rRNA. Sequencing required for speciation.*
Serratia marcescens	16S rRNA	FAM (465-510)	28.92-32.14	*Iwaya A. et al, 2005*	*Species specific 16S rRNA*
Acinetobacter baumannii	bap	HEX (533-580)	31.25-33.60	*De gregorio E. et al, 2015*	
Pseudomonas aeruginosa	oprL	Cy 5 (618-660)	33.45-35.63	*De Vos D. et al, 1997*	
Kingella kingae	rtxA	FAM (465-510)	36.44-37.34	*Lehours P. et al, 2011*	
Klebsiella pneumoniae	rcsA	HEX (533-580)	31.94-36.88	*Derong D. et al, 2015*	
Escherichia coli	yccT	Cy 5 (618-660)	34.84-37.67	*Gadsby N.J. et al, 2015*	
Enterococcus faecalis	ddlE	FAM (465-510)	32.22-36.12	*Micropathology Ltd*	
Enterococcus faecium	ddlE	HEX (533-580)	30.11-33.20		

Organism (bacteria)	Gene	Dye/Detection Filter	LLoD (Ct range)	Original Sequence Citation	Comments
Enterobacter cloacae	OmpA	Cy 5 (618-660)	32.58-32.88		
Enterobacter aerogenes	ramA	FAM (465-510)	35.07-38.25	Micropathology Ltd	*Both genes cover sequences found in E. coli, Shigella, Salmonella, Citrobacter, Klebsiella, Enterobacter, Serratia, Proteus, Morganella, Yersinia, Erwinia*
Enterobacteriaceae spp. #1	rpoB	HEX (533-580)	33.67-34.11		
Enterobacteriaceae spp. #2	16SrRNA	Cy 5 (618-660)	33.60-35.87		
**Organism (fungi)**	**Gene**	**Dye/Detection Filter**	**LLoD (Ct range)**	**Original Sequence Citation**	**Comments**
Aspergillus spp. #1	18S rRNA	FAM (465-510)	37.38-39.97	*Nakano S. et al, 2017*	*All gene targets show specificity for A. fumigatus, A. nidulans, A. niger specifics. Sequencing required for confirmation.*
Aspergillus spp. #2	ITS-1	HEX (533-580)	35.28-37.98		
Aspergillus spp. #3	ITS-2	Cy 5 (618-660)	34.74-38.15		
Candida spp. #1	18SrRNA	FAM (465-510)	31.34-35.23	*Sunao S. et al, 2012*	*Probe is specific for C. albicans, C. parapsilosis, C. tropicalis, C. guilliermondii*
Candida spp. #2	18SrRNA	HEX (533-580)	32.58-36.30		*Probe is specific for C. glabrata*
Candida spp. #3	18SrRNA	Cy 5 (618-660)	36.43-39.03		*Probe is specific for C. krusei*

10 multiplex hydrolysis probe-based assays were used to detect 24 bacterial and 6 fungal gene variants. Multiple gene targets for some organisms or groups of organisms were used to maximise the sensitivity of the tests (indicated by #1, #2, #3). Where the Ct associated with an amplification curve fell within the lower limit of detection range (LLoD), the qPCR was repeated or 16S rRNA/18S rRNA performed for confirmation

Ct: cycle threshold

Table S5: *Investigation of potential confounders.*

**Comparison**	**Coefficient**	**Estimate**	**Std Error**	**p**
**Case vs Control**	Age	-0.0047	0.00029	$<2e^{-16}$
**Case vs Control**	Gender	0.035	0.033	0.3
**Case vs Control**	Ethnicity	-0.339	0.242	0.16
**Case vs Control**	Country	0.089	0.058	0.13
**Case vs Control**	Immunodeficiency	-0.154	1.97	0.94
**Definite Bacterial vs Definite Viral**	Age	0.0033	0.00066	$5.9e^{-7}$
**Definite Bacterial vs Definite Viral**	Gender	-0.021	0.081	0.8
**Definite Bacterial vs Definite Viral**	Ethnicity	-0.902	1.01	0.37
**Definite Bacterial vs Definite Viral**	Country	-0.093	0.171	0.59
**Definite Bacterial vs Definite Viral**	Immunodeficiency	-0.299	0.193	0.12
**Definite Bacterial vs Definite Viral**	Clinical Syndrome	0.119	0.141	0.39

Potential confounding factors were investigated for their impact on the odds of a child being a case vs control and being classified as definite bacterial vs definite viral. The impact, standard error and p value for these are show in the table. Age was the only significant confounder for both comparisons.

Table S6: *Bacterial detections by clinically indicated pathogen tests in DB patients.*

**Pathogen**	**Definite Bacterial**
Bordetella pertussis	6
Borrelia burgdorferi	13
Campylobacter spp.	20
Clostridium perfringens	2
Enterobacter cloacae	12
Enterococcus faecalis	13
Enterococcus faecium	4
Enterococcus spp.	4
Escherichia coli	209
Fusobacterium necrophorum	3
Fusobacterium spp.	1
Haemophilus influenzae (non-type B)	3
Haemophilus influenzae (type B)	1
Haemophilus influenzae (unspecified type)	7
Kingella kingae	6
Klebsiella oxytoca	3
Klebsiella pneumoniae	10
Klebsiella spp.	2
Micrococcus spp.	1
Mycobacterium tuberculosis	10
Mycoplasma pneumoniae	40
Mycoplasma spp.	4
Neisseria meningitidis	30
Prevotella spp	1
Proteus spp.	3
Pseudomonas aeruginosa	28
Pseudomonas spp.	1
Salmonella spp.	26
Staphylococcus - coagulase negative	25
Staphylococcus aureus	74
Streptococcus - alpha haemolytic, no further information	3
Streptococcus Group A (Strep. pyogenes)	30
Streptococcus Group B (includes Strep.agalactiae)	6
Streptococcus Group F (includes anginosus, milleri)	6
Streptococcus pneumoniae	45
Streptococcus viridans group	7
Other Bacteria	40

The frequency of bacterial detection by clinically indicated pathogen tests run locally at each recruitment site in patients assigned a “Definite Bacterial” diagnostic category.

Table S7: *Viral detections by local, clinically indicated pathogen tests by diagnostic category.*

Pathogen	***Definite Bacterial***	***Probable Bacterial***	***Bacterial Syndrome***	***Unknown Bacterial or Viral***	***Viral Syndrome***	***Probable Viral***	***Definite Viral***	***Other Phenotype***
Adenovirus	15	7	2	25	32	4	60	10
Bocavirus	1	0	1	3	0	0	4	0
Coronavirus	2	1	0	2	2	2	11	2
Cytomegalovirus	1	0	1	1	2	2	6	3
Dengue virus	0	0	0	0	1	0	2	0
Enterovirus	5	8	1	35	19	9	55	6
Epstein-Barr Virus	3	4	1	5	9	8	42	4
Hepatitis A virus	0	0	0	0	0	0	8	0
Herpes simplex	0	0	0	2	0	0	3	0
Herpes simplex type 1	1	1	1	4	2	1	15	0
Herpes simplex type 2	0	0	0	0	0	0	1	0
HIV 1	0	0	0	0	0	0	1	1
Human herpesvirus 6	0	0	0	1	2	1	6	3
Influenza A	3	6	0	6	18	5	61	1
Influenza B	3	3	0	4	6	3	38	2
Measles	1	0	0	0	2	0	29	0
Metapneumovirus	0	4	1	3	4	1	9	2
Norovirus	0	2	1	5	4	0	11	4
Other Virus	1	3	0	0	2	3	7	0
Parainfluenza type 1	1	3	0	4	5	0	4	1
Parainfluenza type 2	1	2	0	2	0	0	1	1
Parainfluenza type 3	1	2	1	7	9	1	7	0
Parainfluenza type 4	0	0	0	2	3	0	3	2
Parechovirus	0	0	0	1	1	0	1	1
Parvovirus	0	0	0	0	1	0	10	4
Respiratory syncytial virus	1	6	1	16	23	5	57	3
Rhinovirus	16	21	3	62	40	14	50	16
Rotavirus	4	3	1	1	7	4	19	4
Varicella zoster virus	3	4	10	2	0	0	14	3

The frequency of viral detection by clinically indicated pathogen tests run locally at each recruitment site in each locally-assigned diagnostic category. Viruses listed for patients in the probable viral group were not deemed to explain the full presenting clinical syndrome.

Table S8: *Frequency of pathogen detection in throat swabs by centralized molecular tests in relation to the clinically-assigned diagnostic category.*

Pathogen	Definite Bacterial (n=417)	Probable Bacterial (n=434)	Bacterial Syndrome (n=270)	Unknown Bacterial or Viral (n=425)	Viral Syndrome (n=173)	Probable Viral (n=401)	Definite Viral (n=280)	Other Phenotype (n=362)	Overall Cases (n=2762)	Controls (n=485)
Adenovirus	17	41	14	38	28	26	13	14	191 (6.04%)	10 (2.37%)
Bocavirus	10	23	17	30	12	21	14	11	138 (4.36%)	8 (1.9%)
C.pneumoniae	3	3	0	1	1	2	0	2	12 (0.38%)	3 (0.71%)
Coronavirus 229E	8	9	3	9	5	14	12	2	62 (1.96%)	17 (4.03%)
Coronavirus HKU1	1	1	0	2	2	3	2	3	14 (0.44%)	3 (0.71%)
Coronavirus NL63	5	7	2	4	1	5	5	5	34 (1.07%)	4 (0.95%)
Coronavirus OC43	5	7	6	4	3	13	9	4	51 (1.61%)	4 (0.95%)
Enterovirus	11	7	12	32	18	33	19	6	138 (4.36%)	2 (0.47%)
Influenza A	8	11	10	15	8	28	34	7	121 (3.83%)	4 (0.95%)
Influenza B	3	8	1	7	5	21	26	9	80 (2.53%)	4 (0.95%)
M.pneumoniae	25	6	8	5	1	3	0	2	50 (1.58%)	2 (0.47%)
Metapneumovirus	3	17	3	11	8	10	11	2	65 (2.06%)	5 (1.18%)
Parainfluenza 1	3	5	1	4	5	9	3	2	32 (1.01%)	2 (0.47%)
Parainfluenza 2	1	2	3	6	1	5	2	3	23 (0.73%)	3 (0.71%)
Parainfluenza 3	2	12	5	10	4	18	3	5	59 (1.87%)	4 (0.95%)
Parainfluenza 4	4	3	5	5	1	4	4	4	30 (0.95%)	2 (0.47%)
Rhinovirus	82	101	43	107	39	64	52	58	546 (17.26%)	71 (16.82%)
RSV	4	10	12	31	22	27	31	11	148 (4.68%)	8 (1.9%)
L.pneumophila	0	1	1	1	1	2	0	1	7 (0.22%)	2 (0.47%)
**Total Detections**	**390**	**548**	**292**	**644**	**330**	**616**	**480**	**302**	**1801 (56.94%)**	**158 (37.44%)**

The frequency of each pathogen's detection by CMT in relation to the patient diagnostic category is shown. The overall cases column is the sum of all detections for that virus, expressed as a percentage of all cases tested. The controls column shows detections for that virus expressed as a percentage of all controls tested.

Table S9: *Frequency of virus detection in blood by centralized molecular tests in relation to the clinically-assigned diagnostic category.*

**Pathogen**	**Definite Bacterial (n=569)**	**Probable Bacterial (n=618)**	**Bacterial Syndrome (n=406)**	**Unknown Bacterial or Viral (n=724)**	**Viral Syndrome (n=274)**	**Probable Viral (n=568)**	**Definite Viral (n=454)**	**Other Phenotype (n=562)**	**Overall Cases (n=4175)**	**Controls (n=999)**
Adenovirus	5	24	8	23	20	16	10	7	113 (2.3%)	6 (0.67%)
CMV	16	14	3	17	5	13	13	11	92 (1.87%)	5 (0.55%)
EBV	102	125	60	107	42	84	98	67	685 (13.94%)	146 (16.19%)
Enterovirus	15	10	11	35	20	55	40	9	195 (3.97%)	13 (1.44%)
HHV6a	5	2	1	4	1	5	0	5	23 (0.47%)	5 (0.55%)
HHV6b	148	176	127	185	87	189	93	119	1124 (22.87%)	286 (31.71%)
HHV7	225	270	169	277	100	223	140	237	1641 (33.39%)	528 (58.54%)
Parechovirus	2	5	2	6	4	6	7	3	35 (0.71%)	4 (0.44%)
Parvovirus	37	32	19	31	13	33	23	23	211 (4.29%)	43 (4.77%)
**Total Detections**	**1,110**	**1,316**	**800**	**1,370**	**584**	**1,248**	**848**	**962**	**4119 (83.82%)**	**1036 (114.86%)**

The frequency of each pathogen's detection in relation to the patient diagnostic category is shown. The overall cases column is the sum of all detections for that virus, expressed as a percentage of all cases tested. The controls column shows detections for that virus expressed as a percentage of all controls tested.

***Table S10**: Frequency of bacterial/fungal detection in blood by centralized molecular tests in relation to the clinically-assigned diagnostic category*

Pathogen	***Definite Bacterial (n=567)***	***Probable Bacterial (n=614)***	***Bacterial Syndrome (n=404)***	***Unknown Bacterial or Viral (n=706)***	***Viral Syndrome (n=268)***	***Probable Viral (n=564)***	***Definite Viral (n=448)***	***Other Phenotype (n=549)***	***Overall Cases (n=4120)***	***Controls (n=996)***
A.baumanii	1	6	3	2	1	4	3	4	24 (0.48%)	10 (1.06%)
E.aerogenes	3	2	1	4	0	1	2	0	13 (0.26%)	2 (0.21%)
E.cloacae	5	5	1	2	1	2	2	1	19 (0.38%)	3 (0.32%)
E.coli	8	8	4	4	1	3	1	5	34 (0.69%)	9 (0.95%)
E.faecalis	4	10	2	2	2	2	1	3	26 (0.52%)	6 (0.63%)
E.faecium	8	6	1	3	3	2	2	3	28 (0.56%)	3 (0.32%)
Group A Streptococcus	9	6	3	2	1	0	1	0	22 (0.44%)	5 (0.53%)
Group B Streptococcus	2	6	0	1	2	1	3	1	16 (0.32%)	3 (0.32%)
H.influenzae	1	5	3	3	2	3	3	0	20 (0.4%)	1 (0.11%)
K.kingae	1	3	0	2	0	0	0	0	6 (0.12%)	0 (0%)
K.pneumoniae	9	9	2	6	4	9	3	8	50 (1.01%)	12 (1.27%)
N.meningitidis	26	10	5	5	2	4	2	0	54 (1.09%)	7 (0.74%)
P.aeruginosa	5	6	6	4	5	0	4	3	33 (0.66%)	6 (0.63%)
S.aureus	13	6	2	5	4	9	9	0	48 (0.97%)	15 (1.58%)
S.marcescens	6	11	4	5	4	3	2	9	44 (0.89%)	6 (0.63%)
S.pneumoniae	19	8	3	6	2	0	1	1	40 (0.81%)	3 (0.32%)
Aspergillus Sp.	0	2	0	1	0	0	0	0	3 (0.06%)	0 (0%)
Candida Sp.	0	2	0	1	0	0	0	0	3 (0.06%)	0 (0%)
Total Detections	240	222	80	116	68	86	78	76	483 (9.73%)	91 (9.61%)

The frequency of each pathogen's detection in relation to the patient diagnostic category is shown. The overall cases column is the sum of all detections for that pathogen, expressed as a percentage of all cases tested. The controls column shows detections for that pathogen expressed as a percentage of all controls tested.

***Table S11**: Predictive values of viruses for ruling in or out a DB or PB infection*

PATHOGEN	PREDICTIVE VALUE FOR EXCLUDING DB & PB
**Respiratory Viruses**	
Influenza A	0.820
Influenza B	0.847
RSV	0.887
Parainfluenza 1	0.696
Parainfluenza 2	0.727
Parainfluenza 3	0.707
Parainfluenza 4	0.533
Coronavirus 229E	0.673
Coronavirus NL63	0.526
Coronavirus OC43	0.697
Coronavirus HKU1	0.667
Metapneumovirus	0.640
Adenovirus	0.627
Bocavirus	0.663
Rhinovirus	0.584
Enterovirus	0.839
**Any Detection**	**0.682**
**Blood Viruses**	
Adenovirus	0.695
CMV	0.618
EBV	0.598
HHV6a	0.625
HHV6b	0.634
HHV7	0.603
Parvovirus	0.593
Enterovirus	0.852
Parechovirus	0.767
**Any Detection**	**0.639**

Predictive value for ruling-out a bacterial infection (defined as a DB or PB phenotype patient), in relation to all other combined infection categories if a virus was found in the throat swab or in the blood. The table shows the predictive value for each virus in the panel, and the combined values when the respiratory panel or the blood viruses were taken together. Predictive values were calculated by dividing the number of patients with a diagnostic category other than definite bacterial and probable bacterial in whom each virus was detected by the total number of patients in whom that virus is detected.

Supplementary Figures

Figure S1*: Odds ratios for molecular pathogen identifications in combined Definite and*

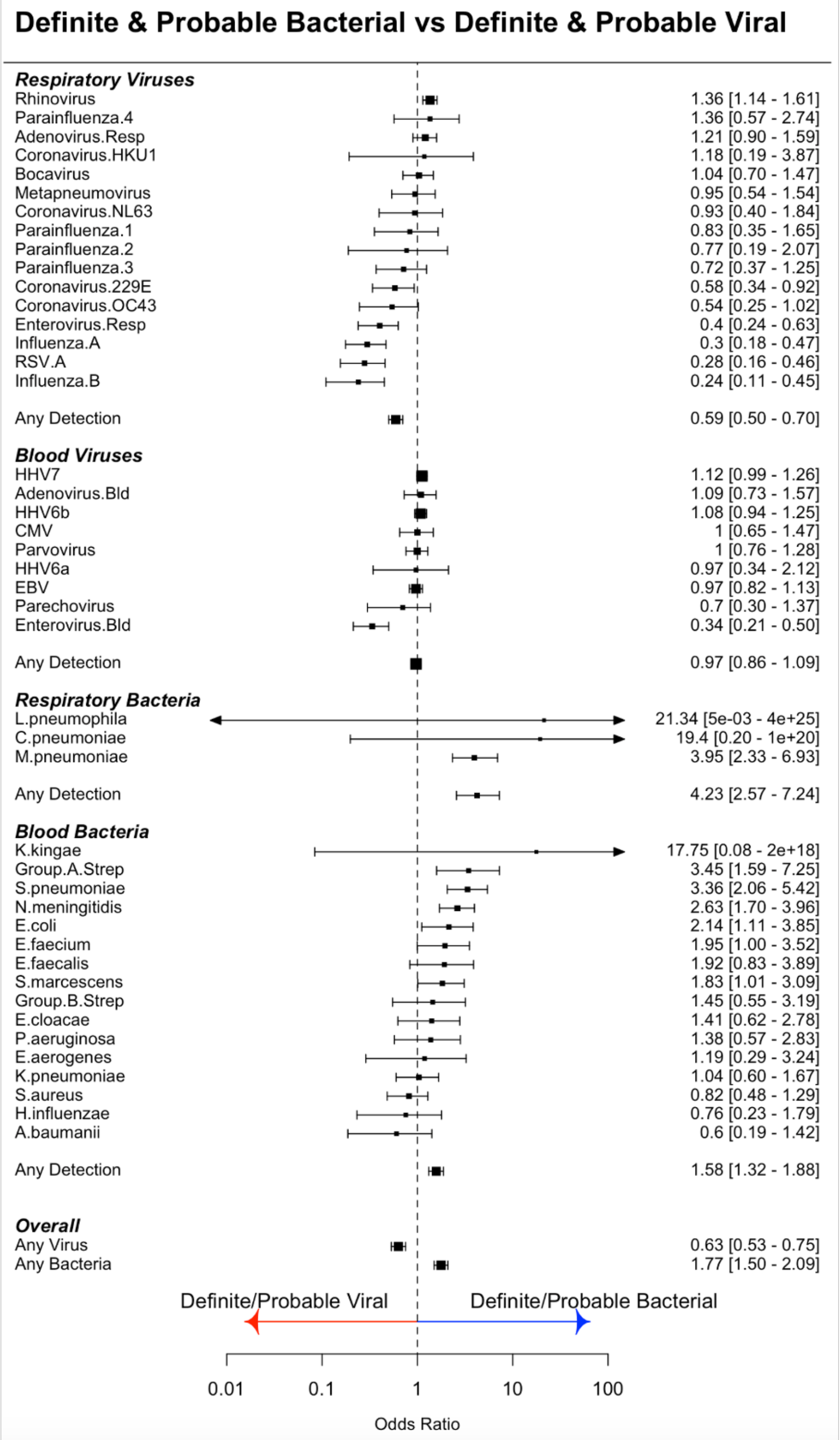

Probable Bacterial groups compared to Definite and Probable Viral groups

Odds ratios (OR) of a positive detection of a target in patients assigned as Definite or Probable Bacterial diagnostic category compared with those in the Definite or Probable Viral categories. OR above 1 indicates more frequent detection in a patient with bacterial infection and OR below 1 indicates increased detection in those with viral infection. Whiskers around the OR represent the 95% confidence interval.

Figure S2: *Odds ratios for pathogen detection by CMT in cases vs controls in children under and over 1 year of age.*

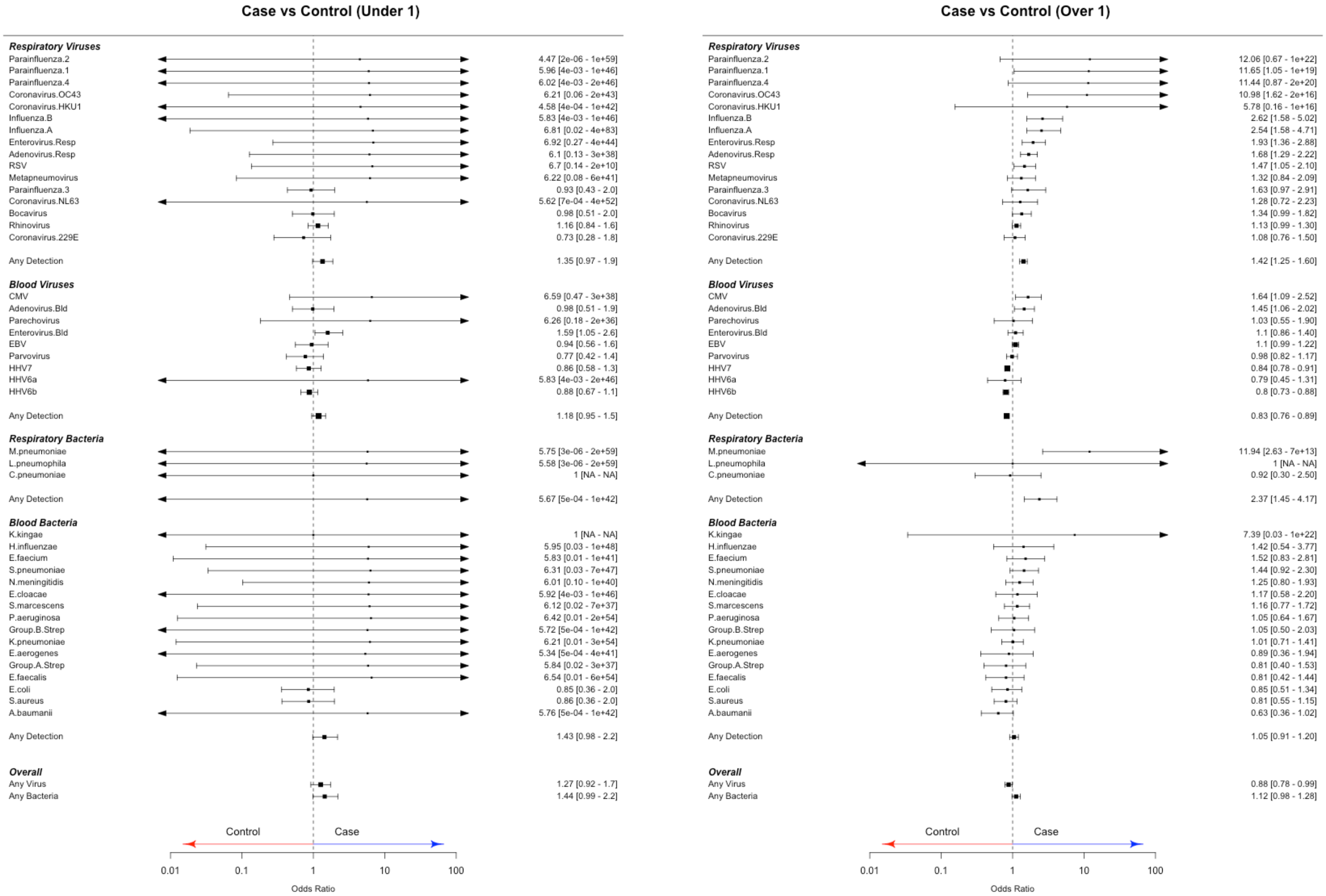

Figure S3: *Odds ratios for pathogen detection by CMT in definite bacterial vs definite viral groups in children under and over 1 year of age.*

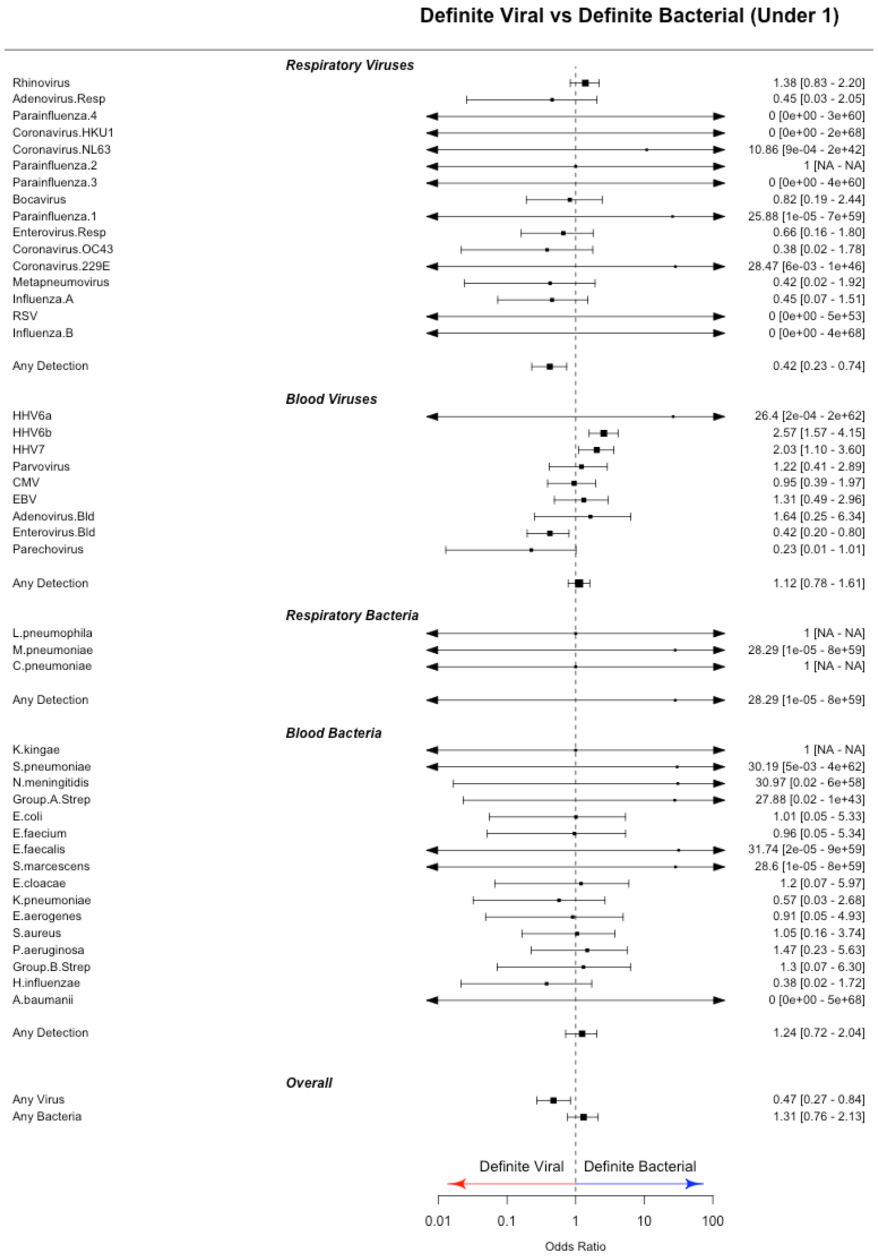

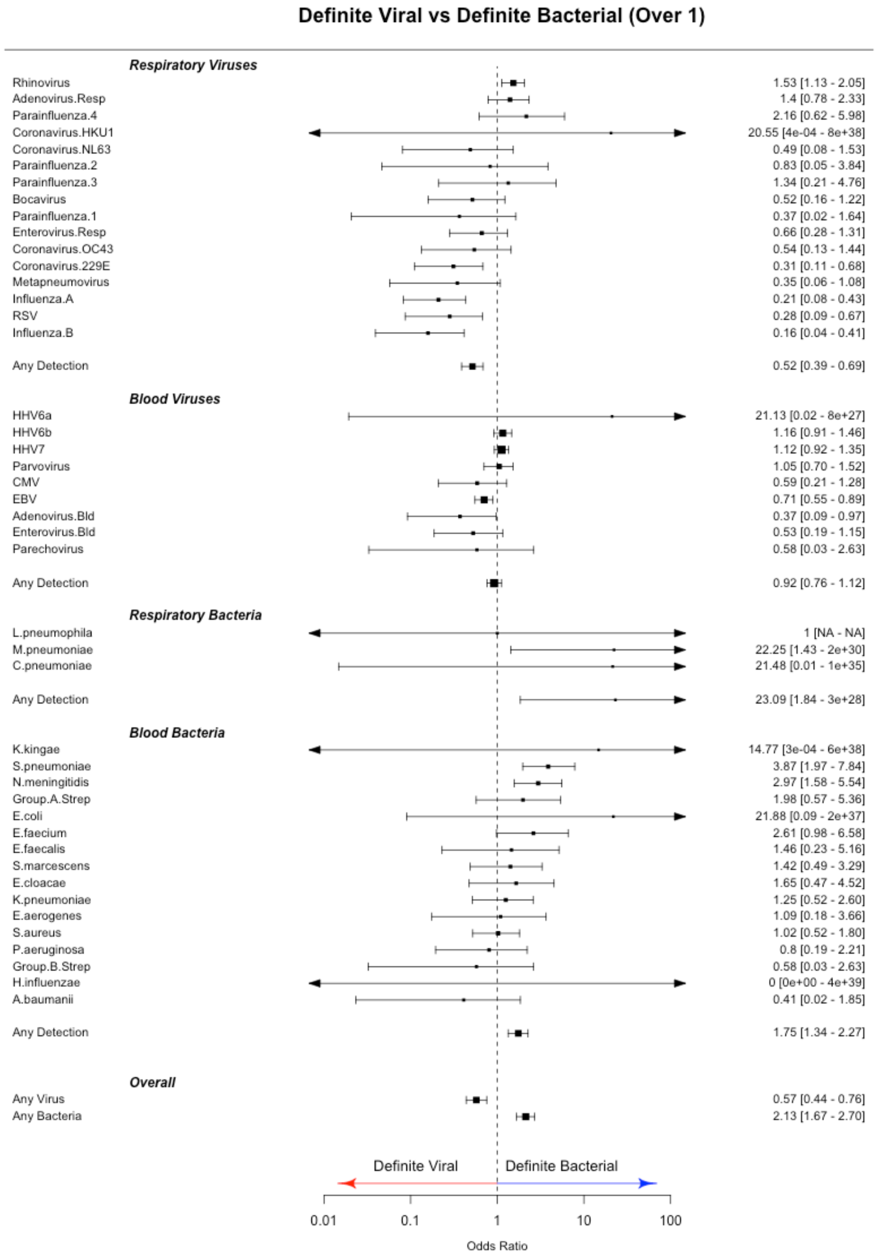

***Figure S4**: Quantitative molecular detection by increasing viral load quartile – blood viruses in relation to cases vs controls*

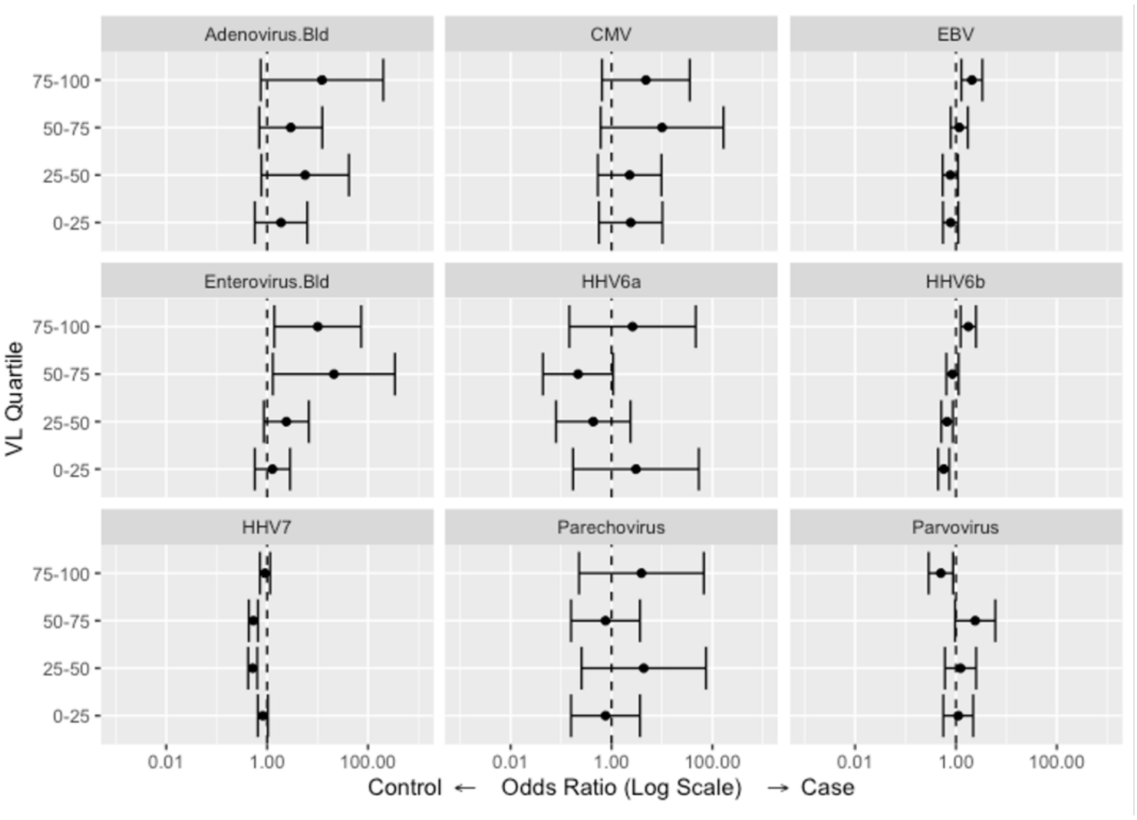

Odds ratio with 95% CIs for detection of blood viruses in cases versus controls at increasing levels of viral load.

***Figure S5**: Quantitative molecular detection by increasing viral load quartile – blood viruses in relation to Definite Viral vs Definite Bacterial diagnostic categories.*

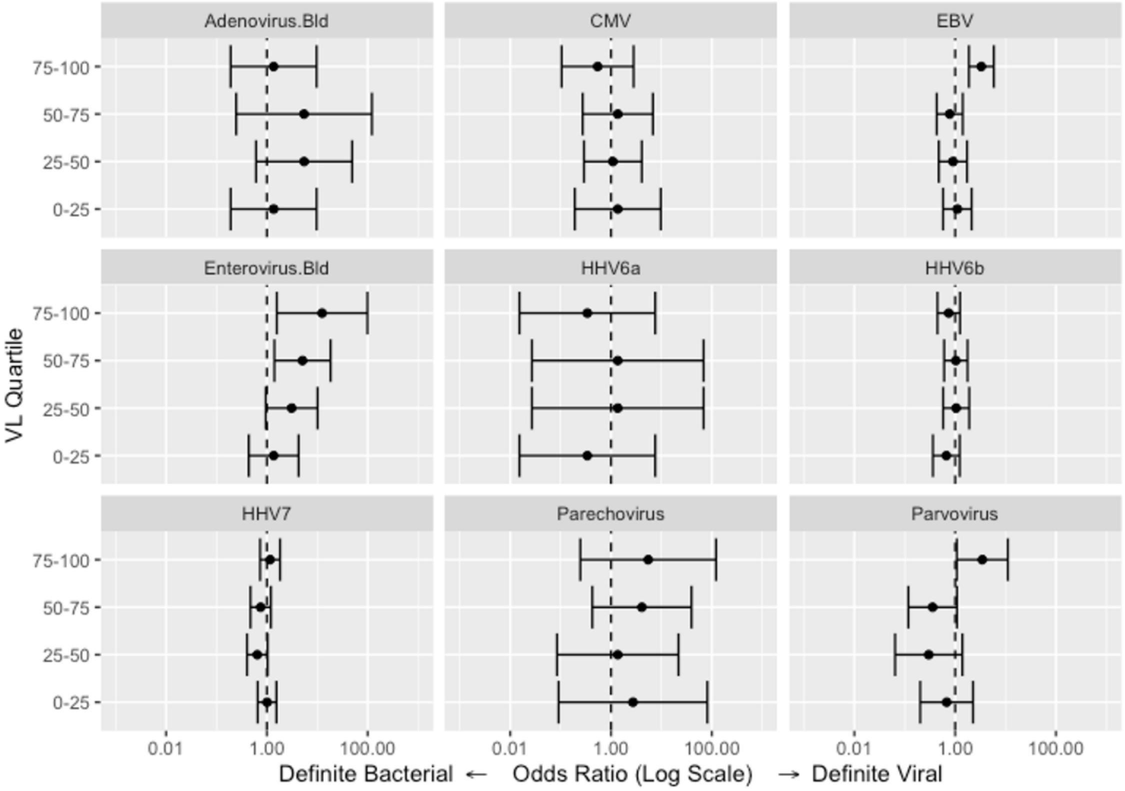

Odds ratio for detection of blood viruses in Definite Viral versus Definite Bacterial cases, at increasing levels of viral load.
